# Decadal (2006-2018) dynamics of Southwestern Atlantic’s largest turbid zone reefs

**DOI:** 10.1371/journal.pone.0247111

**Published:** 2021-02-22

**Authors:** Carolina D. Teixeira, Pamela M. Chiroque-Solano, Felipe V. Ribeiro, Lélis A. Carlos-Júnior, Leonardo M. Neves, Paulo S. Salomon, Leonardo T. Salgado, Ludmilla N. Falsarella, Gabriel O. Cardoso, Lívia B. Villela, Matheus O. Freitas, Fernando C. Moraes, Alex C. Bastos, Rodrigo L. Moura

**Affiliations:** 1 Instituto de Biologia and SAGE-COPPE, Universidade Federal do Rio de Janeiro, Rio de Janeiro, RJ, Brazil; 2 Laboratório de Ecologia Aquática e Educação Ambiental, Universidade Federal Rural do Rio de Janeiro, Três Rios, Rio de Janeiro, RJ, Brazil; 3 Instituto de Pesquisas Jardim Botânico do Rio de Janeiro (JBRJ), Rio de Janeiro, RJ, Brazil; 4 Instituto Meros do Brasil, Curitiba, Paraná, Brazil; 5 Universidade Federal do Espírito Santo, Vitória, Brazil; Secretariat of the Pacific Community, NEW CALEDONIA

## Abstract

Tropical reefs are declining rapidly due to climate changes and local stressors such as water quality deterioration and overfishing. The so-called marginal reefs sustain significant coral cover and growth but are dominated by fewer species adapted to suboptimal conditions to most coral species. However, the dynamics of marginal systems may diverge from that of the archetypical oligotrophic tropical reefs, and it is unclear whether they are more or less susceptible to anthropogenic stress. Here, we present the largest (100 fixed quadrats at five reefs) and longest time series (13 years) of benthic cover data for Southwestern Atlantic turbid zone reefs, covering sites under contrasting anthropogenic and oceanographic forcing. Specifically, we addressed how benthic cover changed among habitats and sites, and possible dominance-shift trends. We found less temporal variation in offshore pinnacles’ tops than on nearshore ones and, conversely, higher temporal fluctuation on offshore pinnacles’ walls than on nearshore ones. In general, the Abrolhos reefs sustained a stable coral cover and we did not record regional-level dominance shifts favoring other organisms. However, coral decline was evidenced in one reef near a dredging disposal site. Relative abundances of longer-lived reef builders showed a high level of synchrony, which indicates that their dynamics fluctuate under similar drivers. Therefore, changes on those drivers could threaten the stability of these reefs. With the intensification of thermal anomalies and land-based stressors, it is unclear whether the Abrolhos reefs will keep providing key ecosystem services. It is paramount to restrain local stressors that contributed to coral reef deterioration in the last decades, once reversal and restoration tend to become increasingly difficult as coral reefs degrade further and climate changes escalate.

## Introduction

Tropical reefs are declining rapidly due to ocean warming, associated with mass coral bleaching and mortality [1], and also by local stressors such as degrading water quality and overfishing [2, 3]. Heat stress and eutrophication also elicit diseases and predator outbreaks, which are major causes of coral mortality [4]. In the last four decades, global coral cover declined between 50 and 75% [5]. Such widespread degradation impairs biomineralization, reduces shoreline protection, fisheries production, and other ecosystem services, with profound impacts to the livelihoods of millions of people [6]. Reversal and restoration are urgently needed and will become increasingly difficult as coral reefs degrade further [7].

Corals are foundation organisms that engineer most of the structural complexity of tropical reefs [8]. The functioning of coral holobionts, which host a diverse assemblage of microorganisms, is optimal under temperature and nutrient levels near their upper thresholds, making corals highly vulnerable to environmental stress (e.g. [9]). Reduction of live coral cover and diversity are among the most evident consequences of declining reef ecosystems [10, 11]. The temporally-persistent replacement of corals by non-building organisms, known as phase shift [10, 12], is associated with lower structural complexity and decreased community level-diversity [13], as well as to microbialization and diminished secondary production [14, 15]. After the sharp decline of Caribbean reefs in the 1980’s-1990’s, understanding the patterns and drivers of phase shifts became a major theme in tropical reef ecology [7, 16]. However, the scarcity of baselines and time series [17], as well as major geographic data gaps, still impedes a thorough understanding of this phenomenon, its context-dependency [7, 18], and the multiplicative or antagonistic effects of global and local drivers upon coral decline [5, 19]. The overall complexity of reef systems and the wide array of environmental conditions under which they are able to persist adds to the complexity of understanding phase shifts.

The so-called marginal reefs [20] thrive across the world in turbid-zones (high nutrient and/or sedimentation levels) or in subtropical latitudes/deeper water (colder, eutrophic and/or light-limited settings). The global area of marginal reefs may be as large as that of oligotrophic reefs [21–23], but mapping and baselines are far from comprehensive, because many of these reefs are not readily accessed by remote sensing or divers.

Albeit dominated by fewer species with adaptations to conditions that are suboptimal to most coral species, marginal reefs may have significant coral cover and growth [20] and may function as refugia during thermal anomalies [24–26]. The dynamics of marginal reefs may deviate from that of archetypical oligotrophic tropical reefs [25, 27], but it has been assessed less often in both the Pacific [28–30] and in the North Atlantic [31, 32]. The Southwestern Atlantic (SWA) is a major geographic gap (but see [33, 34]), and this data poor scenario leads to contrasting conclusions about their potentially higher (e.g. [35]) or lower resistance (e.g. [36]) to environmental changes [37].

The SWA encompasses the most widely distributed turbid zone reefs in the Atlantic [23], between 5°N to 24°S, and seconds the Caribbean as a biodiversity center in the Atlantic [38]. Here, we present the largest and longest time series of data ever compiled for SWA largest reef complex (Abrolhos), covering two different habitats in five sites that are under a gradient of anthropogenic pressures, longshore and cross-shelf oceanographic forcing. Besides exploring changes in the benthic assemblage structure, we examined whether fast-growing fleshy organisms (e.g. macroalgae, turf, benthic cyanobacteria mats) tended to substitute coral cover in two different habitats (pinnacles tops and walls). Our main goal was to assess the dynamics of the benthic assemblage. Specifically, we aimed at the following questions: 1) How did benthic cover change among habitats and reef sites between 2006 and 2018? 2) Were there regional and/or site-specific dominance-shift trends? 3) How much did the differences in life-history of the most abundant organisms (e.g. corals, macroalgae, zoanthids) influence assemblage dynamics?

## Materials and methods

### Study area

**Fig 1:**
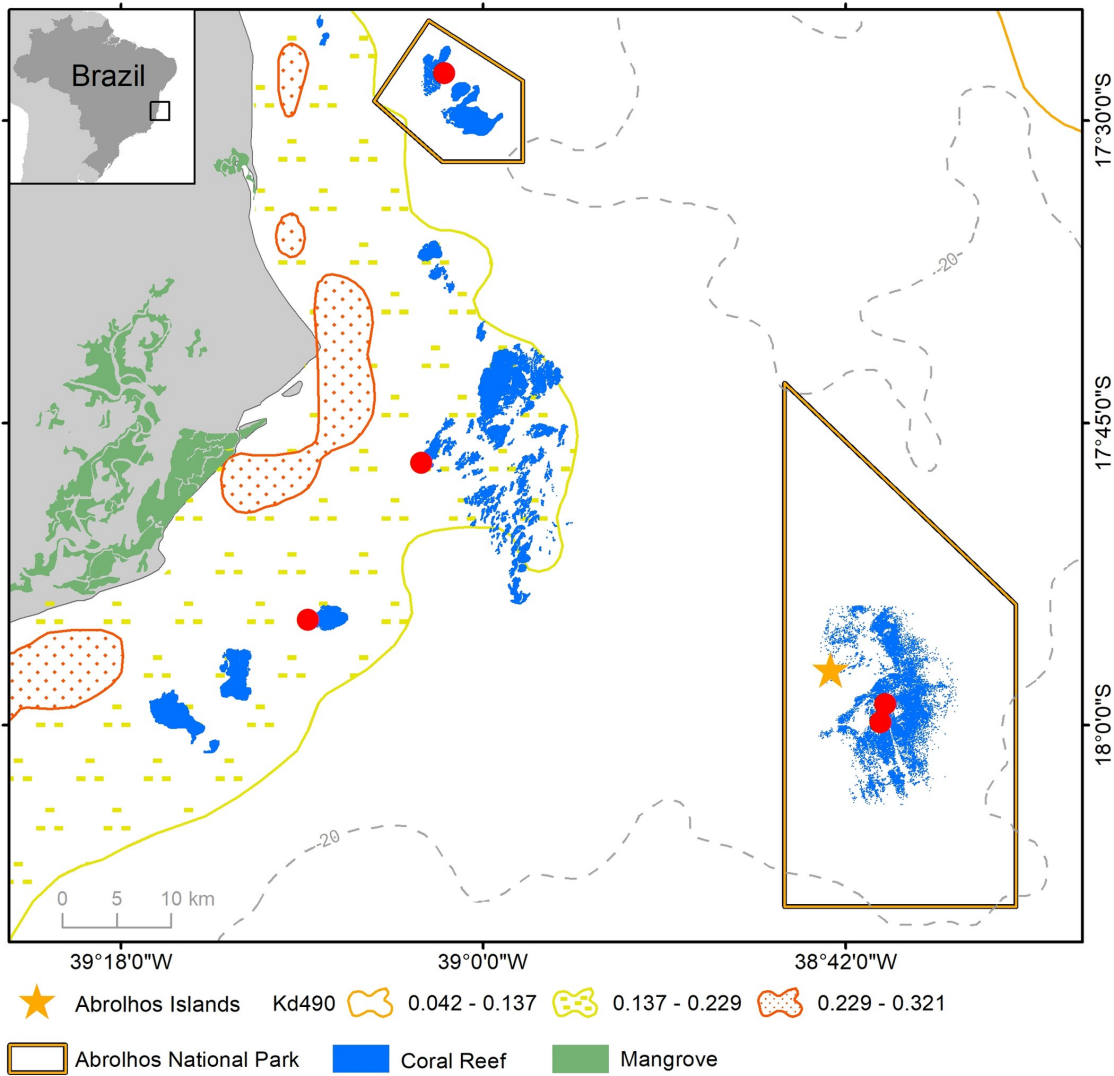
Map of the study region (Abrolhos reefs, Brazil). (A) Location of the five reef sites sampled between 2006 and 2018. Turbidity (Kd490) refers to winter averages of 2018; note higher values associated with nearshore river mouths, the cross-shelf-gradient, and the lower values associated with the northernmost site (TIM), similar to those on the outer arc. (B) Isolated pinnacles under lower turbidity and typical of the outer arc; (C) Coalesced pinnacles under greater turbidity and typical of the nearshore arc. The boat visible in B and C measures 15 m. Site codes: TIMB = Timbebas Reef; PLES = Pedra de Leste Reef; SGOM = Sebastião Gomes Reef; PAB = Parcel dos Abrolhos Reef. Photos by R. L. Moura and Fernando C. Moraes. Map drawn by the authors using ArcGIS 10.6 (www.esri.com) and based on geospatial data freely available from Marinha do Brasil (www.marinha.mil.br/dhn/).

**Fig 2:**
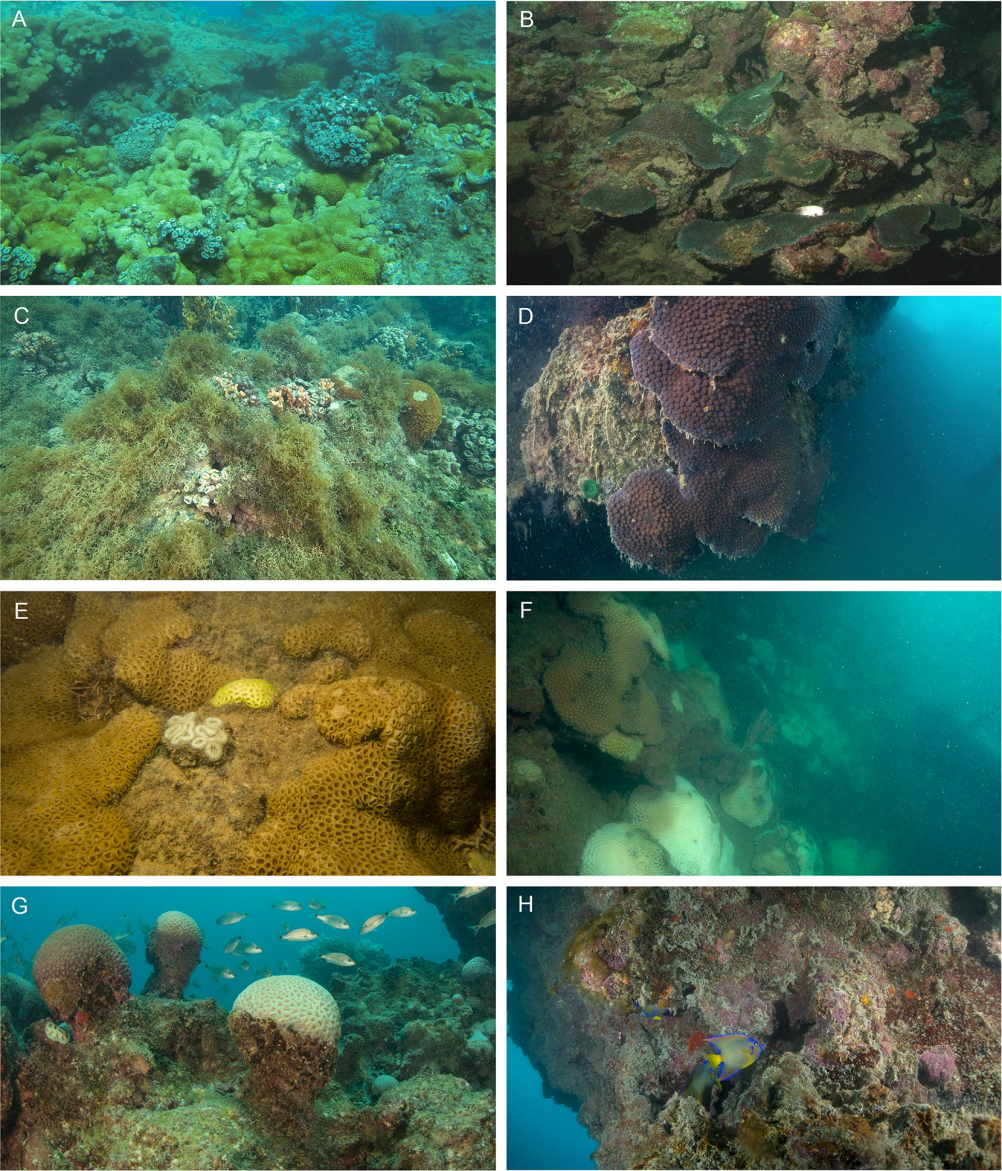
Underwater photographs of tops and walls of each reef sampled in the Abrolhos reefs, Brazil. (left and right column, respectively): A and B) Timbebas (TIMB); C and D) Pedra de Leste (PLES); E and F) Sebastião Gomes (SGOM); G and H) Parcel dos Abrolhos (PAB). Photos taken by the authors.

**Fig 3:**
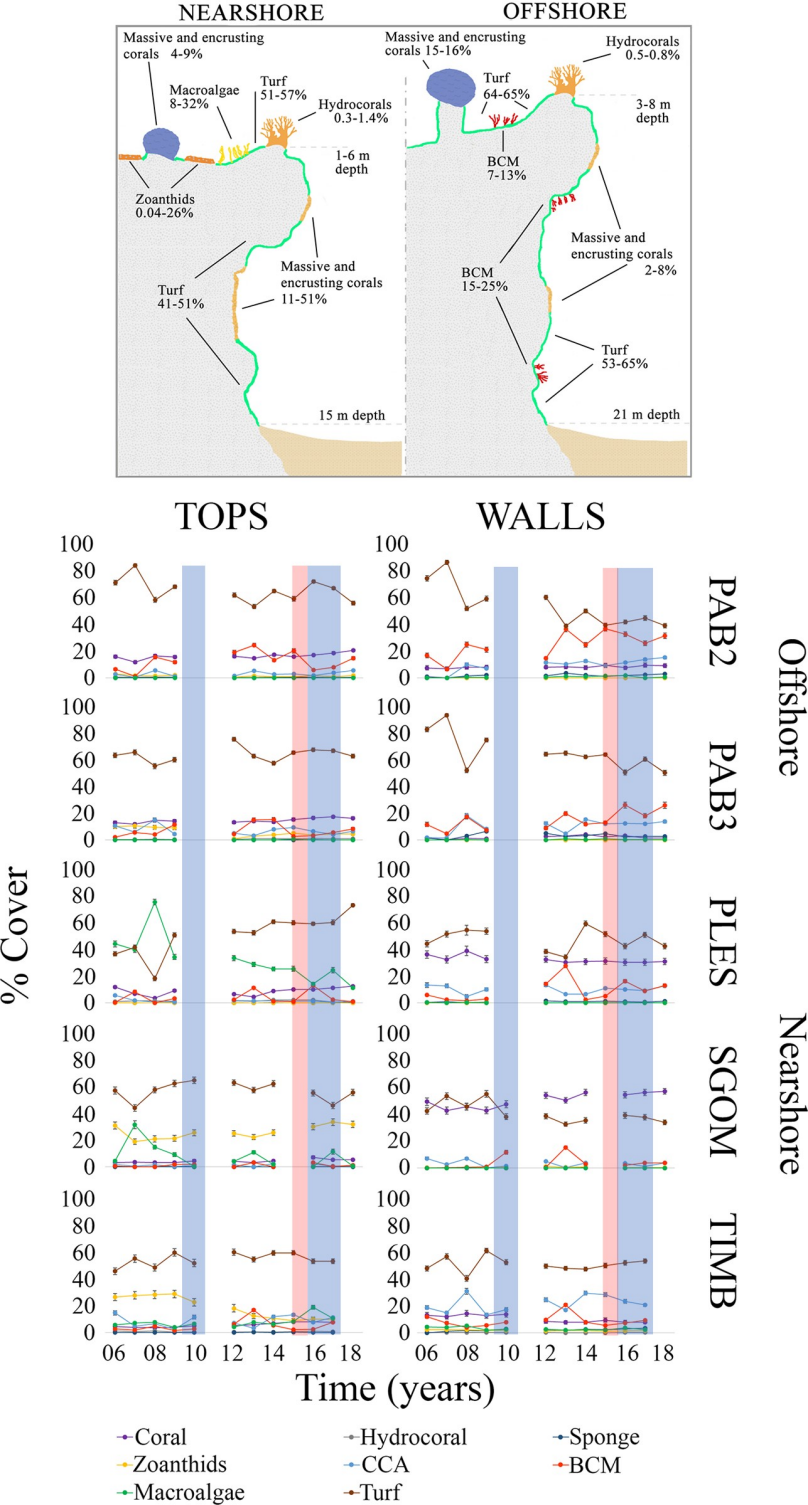
Pinnacles’ morphology and benthic cover across sites and years in the Abrolhos reefs, Brazil. The upper panel shows the mushroom-shaped morphology of the pinnacles, with well-lit, shallow and expanded flat tops and steep shaded walls. The lower panel shows the relative cover of the nine most abundant sessile groups in the five sites (see Fig 1 for site codes) and two habitats sampled between 2006 and 2018. Bars represent Standard Errors. CCA = crustose calcareous algae, BCM = benthic cyanobacterial mats. Blue shades represent the two major thermal anomalies associated with coral bleaching and red shades represent the Fundão Dam collapse in the Doce River basin.

The Abrolhos Bank (16°40’, 19°40’S—39°10’, 37°20’W) is a 46,000 km² shallow water (<70 m depth) enlargement of the Eastern Brazilian shelf that encompasses the largest and richest SWA coral reefs [39]. Emerging and quasi emerging reefs are distributed in two arcs (nearshore and offshore, 10–20 km and 70 km from the shoreline, respectively), and are surrounded by soft sediments, rhodoliths and low-lying mesophotic reefs [22]. We sampled three reefs in the nearshore arc, where pinnacles often coalesce as larger continuous banks [39], and two reefs in the offshore arc (Fig 1). Reef structures occur as oddly shaped pinnacles with 1–50 m diameters with expanded, shallow (<10 m depth) and relatively flat tops, and steep shaded walls that reach up to 25 m depths [40]. Such reef morphology creates two distinct habitats, tops and walls, which occur in close proximity [33, 39] (Figs 2 and 3 and S1 Text). Turbidity and sedimentation levels are among the world’s highest on living reefs [41], and present marked seasonality, cross-shelf and longshore gradients, which are associated with continental sourcing, transport and winter-storm resuspension [42]. During our study, turbidity derived from remote sensing (light attenuation coefficient at 490 nm, Kd490) was minimum on offshore reefs (Parcel dos Abrolhos, 0.076 m^-1^ yearly average) and in the northernmost coastal reef (Timbebas, 0.079 m^-1^), and maximum on the other coastal reefs (Sebastião Gomes and Pedra de Leste, 0.151 and 0.162 m^-1^, respectively) (S2 Text). Terrigenous sediments are absent offshore but represent a large fraction of nearshore sediments (36–49%). Salinity is relatively high (>37) when compared to most Atlantic and Indo-Pacific reefs. Two major sea surface temperature (SST) anomalies associated with anomalous heat stress and mass coral bleaching [26, 43] were recorded during the study period, in 2010 and 2016–2017 (S2 Text).

A summary of the regional historical climatic and anthropogenic stressors, together with management regimes, is provided in a separate file (S2 Text). An exploratory analysis of the potential drivers of benthic assemblage structure and dynamics is also provided as supporting materials (S2 Text).

### Sampling and annotation

Sessile benthic cover was sampled during austral summers between 2006 and 2018, using 100 fixed photo-quadrats (0.7 m^2^ each) per year. Sampling units were randomly disposed and marked with metal pins at each site (n = 5) in the first year, 10 on pinnacles’ tops (mean depth = 5.1 ± 2.2m) and 10 on walls (11.8 ± 5m). The monitored nearshore sites included two unprotected reefs under the highest turbidity levels, Pedra de Leste (PLES) and Sebastião Gomes (SGOM), the latter near a dredging disposal area (~12 km), as well as one no-take but poorly enforced reef with much lower turbidity, Timbebas (TIMB). Offshore, we sampled two no-take sites (PAB2 and PAB3) within the Parcel dos Abrolhos reef. Due to logistical and funding constrains, sampling was not carried out in 2011; sites PLES, PAB2 and PAB3 were not sampled in 2010; SGOM was not sampled in 2015; and TIMB was not sampled in 2018.

Images were annotated semi-automatically with the deep neural network provided by the CoralNet platform [44], using an 80% confidence threshold (label accuracy: 95.4%, functional group accuracy: 96.9%, fraction above threshold: 53%). Relative cover was estimated from the identification of benthic organisms below 30 random points distributed in each image (one photo-quadrat = a mosaic of 15 high resolution close-up images). Organisms were identified at nine broad taxonomic or functional groups and categorized either as slower-growing longer-lived reef builders [corals, crustose calcareous algae (CCA) and hydrocorals] or their faster-growing shorter-lived antagonists [(frondose macroalgae, turf, benthic cyanobacteria mats (BCM), zoanthids, sponges and “other organisms” (OO)].

### Ethics statement

Permits for fieldwork were granted by the Instituto Chico Mendes de Conservação da Biodiversidade (SISBIO#65055–2). The study did not involve collection of specimens nor any kind of animal sacrifice, and therefore did not require approval by any other specific committee.

### Data analyses

Once habitat is the main source of community variation in the oddly-shaped Abrolhos’ pinnacles [26, 33, 40], analyses were carried out separately for tops and walls. An exploratory PERMANOVA [45] confirmed that habitat had more explanatory power (pseudo-F: 67.04; r^2^: 0.266) than site (pseudo-F: 10.98; r^2^: 0.174), and also evidenced the strong interaction between habitat and site (pseudo-F: 10.21; r^2^: 0.162) (S1 Text). A permutational analysis of multivariate dispersions (PERMDISP [46]) also confirmed homogeneous dispersion of samples within each habitat (S1 Text). A principal component analysis (PCA) on Euclidean distances for benthic cover data was used to investigate variance across years and sites. Vectors based on Pearson correlations >0.4 were overlaid in the two-dimensional ordination space to verify which taxa were associated with spatial and temporal changes in the overall community structure [47]. Temporal variation was assessed by regressing the benthic gradient (first and second principal components) against sampling years for each habitat and site.

Annual changes in benthic cover were examined by comparing values obtained in a given year (cover_(t)_) with those obtained in the previous year (cover_(t-1)_), using the logarithm of the relative change cover [log(cover_(t)_/cover_(t-1)_)]. Community-level stability was assessed with two complementary measurements, synchrony (φ) and coefficient of variation (CV) following Lamy et al. [48]. Community-level cover at time t [Cover_s_ (*t*)] was represented as Σ^S^_i = 1_ Cover_i_ (*t*), where Cover_i_ (*t*) denotes cover at time *t* for species i, and *S* represents the aggregate multispecies-level, with μ_i_ and σ^2^_i_ as its respective mean and variance with respect to the species. The synchrony term of community stability (φ_*S*_) [49] was calculated as φ_*S*_ = σ^2^_S_/(Σ^S^_i = 1_ σ_i_)^2^. This term quantifies the proportion of the variance at the community-level with respect to the maximum variance (σ^2^_S_), *i*.*e*., community-level variance when population-level variables of all species are perfectly correlated over time. The synchrony is standardized between 0 (perfect asynchrony) and 1 (perfect synchrony). Furthermore, CV at community-level *S*(CV_S_) was calculated following Thibaut and Connolly [50], as σ_S_/μ_S_, where μ_S_ and σ^2^_S_ are the mean and variance with respect to the community-level. Note that these statistics are not time-dependent.

The interaction between the cover of corals and their competitors was assessed with separate Phase Shift Indices (PSI) calculated following Bruno et al. [51] for five non-building organisms, for each year and site (turf and BCM on tops and walls; macroalgae and zoanthids on tops, and sponges on walls). The PSI is the first component (PC1) from a PCA on the correlation matrix between the inverse of coral cover and each non-building organism cover. Values of PSI were allocated in six categories according to how strongly in favor or in detriment of corals the shift occurred: +++ = stronger coral loss toward competitor (PSI range from 2.469 to 1.647); ++ = medium coral loss (1.646 to 0.826); + = weaker coral loss (0.825 to 0.005);- = weaker coral gain toward competitor (0.004 to -0.817); -- = medium coral gain (-0.818 to -1.638); --- = stronger coral gain (-1.639 to -2.459). Analyses were carried out in R v. 3.6 environment [52]. Graphical outputs were generated with factoextra [53].

## Results

Over the study period, offshore pinnacles’ tops were dominated by turf (53–84%), corals (12–21%) and BCM (1–25%), and by turf (18–73%), macroalgae (0–75%) and zoanthids (0–34%) nearshore (Fig 3 and S1 Table). Walls were dominated by turf (33–62%) and corals (8–58%) nearshore, and by turf (39–93%) and BCM (5–37%) offshore. Higher coral covers were recorded in nearshore walls (8–58%), followed by offshore (12–21%) and nearshore (3–13%) tops, and offshore walls (0–10%) (Fig 3 and S1 Table).

**Fig 4:**
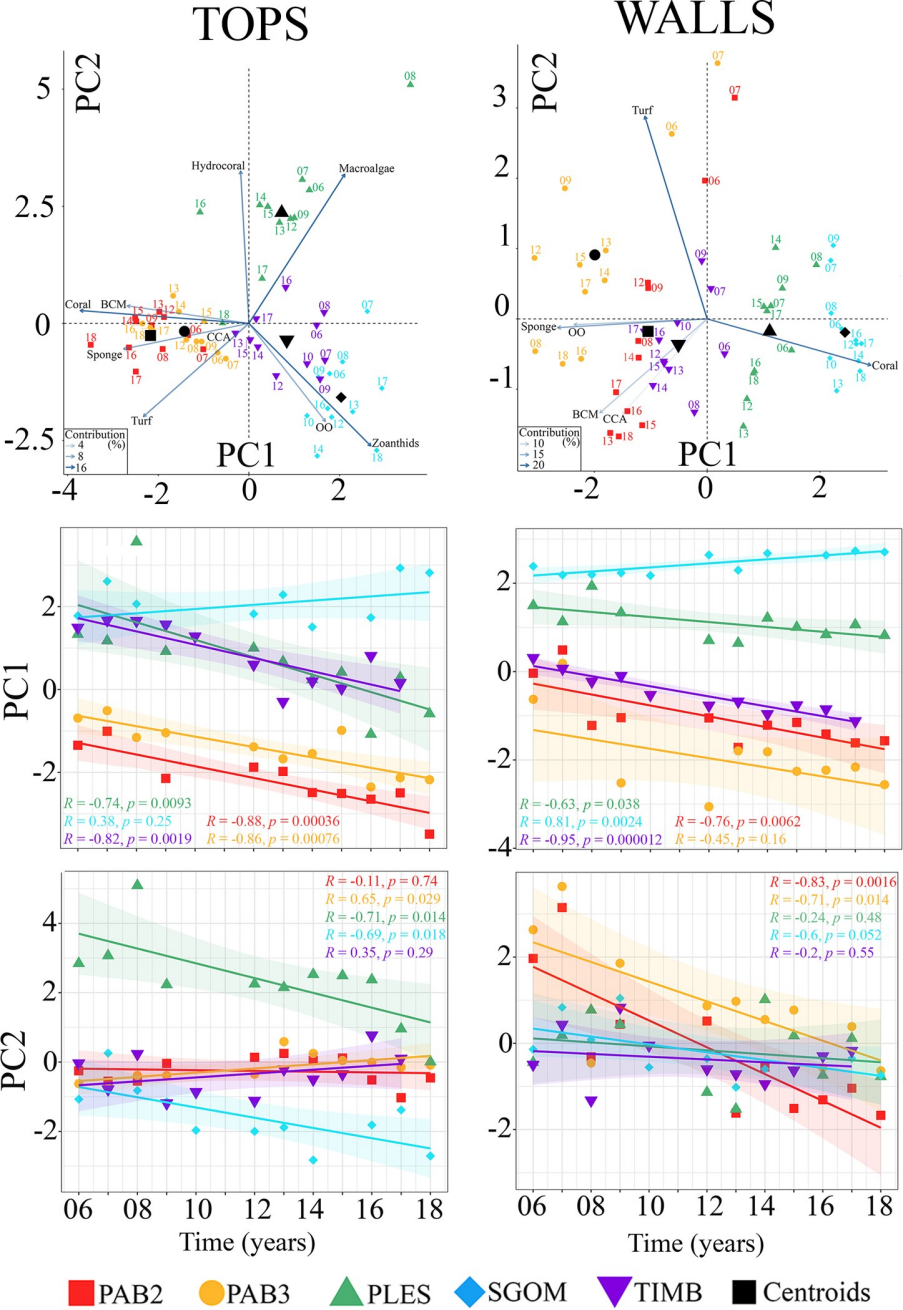
Principal components analyses with all samples for pinnacles’ tops (left) and walls (right). Upper panels show the ordination diagrams, where the numbers above each symbol correspond to the sampling years (TOPS: PC1 = 33.7%, PC2 = 25%, WALLS: PC1 = 48.1%, PC2 = 21.3%). The mid and lower panels show regression of samples’ scores with time for PC1 and PC2, respectively. See Fig 1 for site codes. CCA = crustose calcareous algae, BCM = benthic cyanobacterial mats.

For pinnacles’ tops, the first PCA axis (PC1) evidenced a clear separation between offshore (PAB2 and PAB3) and nearshore reefs (PLES, SGOM and TIMB) (Fig 4), the former associated with higher cover of corals, BCM, turf and sponges, and the latter with macroalgae and zoanthids. The second PCA axis (PC2) discriminated PLES from SGOM, the former associated with higher covers of hydrocorals and macroalgae and the latter with zoanthids and OO (Fig 4). The higher hydrocoral cover nearshore included *Millepora nitida* (Verril, 1868), which was not recorded offshore. Temporal variation was lower offshore and tended to vary mostly along PC1 for offshore tops, and along PC2 for nearshore tops. For walls, such cross-shelf discrimination was also associated with PC1, but samples from TIMB were more similar to those from offshore reefs (Fig 4). Nearshore samples from SGOM and PLES walls were associated with high coral covers (Fig 4) dominated by *Montastraea cavernosa* (Linnaeus, 1767). Temporal change in tops was overall smaller than in walls. Offshore and nearshore tops’ samples were more associated with PC1 and PC2, respectively. Temporal change in walls was largely associated with turf and BCM, which presented opposite trends (Fig 4), and nearshore walls presented lower temporal change than offshore ones. Walls’ samples from 2006 to 2009 presented higher scores on PC2, except for PLES (Fig 4). Although we recorded relevant temporal variation, the overall spatial structure was preserved.

**Fig 5:**
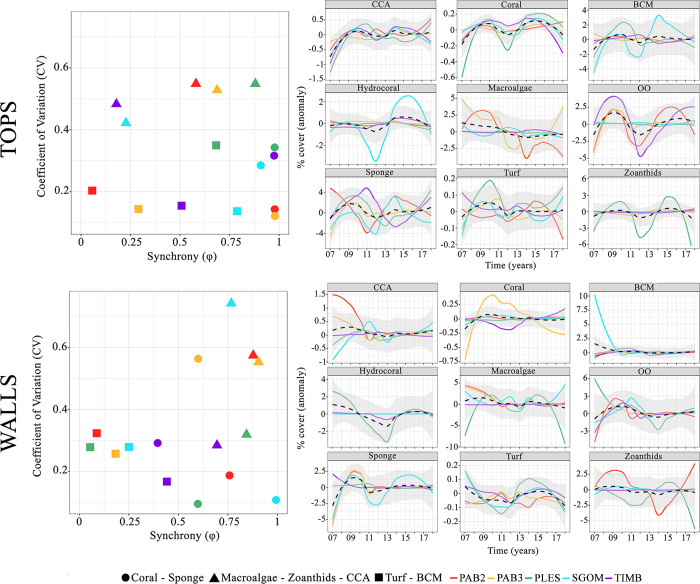
Community stability. Coefficient of variation vs. community’s synchrony (φ) recorded at tops and walls for each site (left). Slower-growing longer-living and faster-growing ephemeral organisms are denoted by circles and squares, respectively, and organisms with intermediate life histories by triangles. The smooth curves (right) represent the cover dynamics of each organism category at each site (anomalies, log-ratio transformed). The black dotted line and grey bands represent the global mean and standard deviation, respectively. BCM = benthic cyanobacteria mats, CCA = crustose calcareous algae, OO = other organisms. See Fig 1 for site codes.

Considering all strata (sites x habitats), dominant organisms presented less variability than rarer ones (Fig 5 and S1 Table). Nearshore tops were more variable than offshore ones due to the shifted dominance from macroalgae to turf in PLES, especially after 2010, and also due to the steadily increasing and decreasing zoanthid cover in SGOM and TIMB, respectively (see Fig 3). Nearshore and offshore walls were dominated by turf, but after 2010 BCM presented higher contributions offshore (see Fig 3). In all strata, stability was associated with the life history of the dominant group (Fig 5). On tops, shorter-lived faster-growing groups were less synchronous (φ = 0.06 ~ 0.88) than corals and sponges, which were less variable (*CV* = 0.12 ~ 0.34) and more synchronous (φ = 0.91 ~ 0.98), especially offshore (PAB2 and PAB3, Fig 5). On walls, long-lived organisms were less variable (*CV* = 0.09 ~ 0.56) and more synchronous (φ = 0.4 ~ 0.99) than shorter-lived ones (*CV* = 0.17 ~ 0.32, φ = 0.05 ~ 0.44). Conversely, organisms with intermediate life histories (macroalgae-zoanthids-CCA) were more synchronous (φ = 0.69 ~ 0.9) but varied the most on walls (*CV* = 0.28 ~ 0.74) (Fig 5).

**Fig 6:**
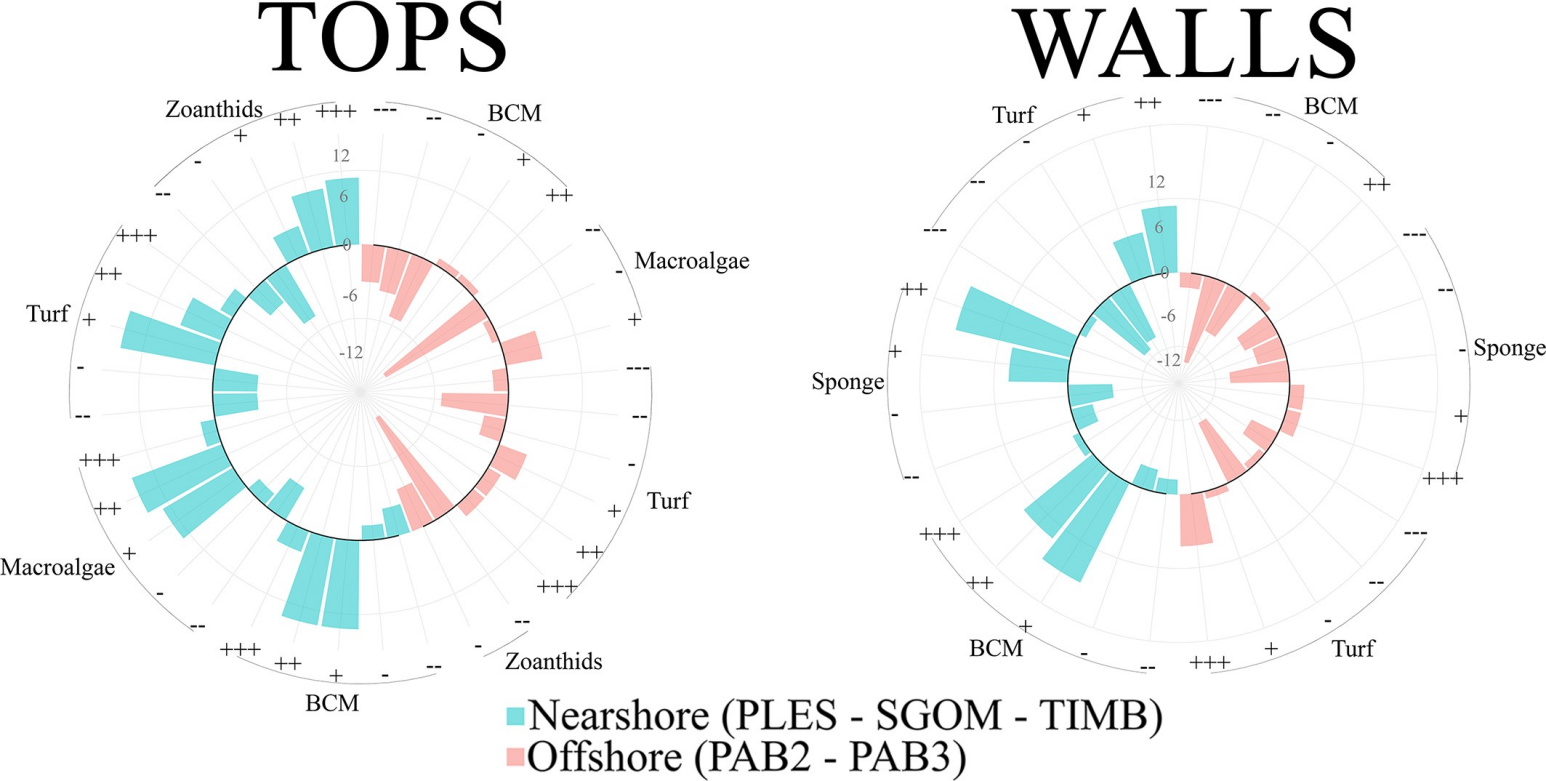
Phase Shift Index (PSI) variation. Frequency of PSI values in pinnacles’ tops (A) and walls (B). Bars inside and outside the inner circle represent negative and positive PSI values, respectively. The radial scale represents the magnitude of the changes expressed as counts of PSI values for each attractor: +++ = higher coral loss toward competitor; ++ = medium coral loss; + = lower coral loss;- = lower coral gain toward competitor; -- = medium coral gain; --- = higher coral gain.

**Fig 7:**
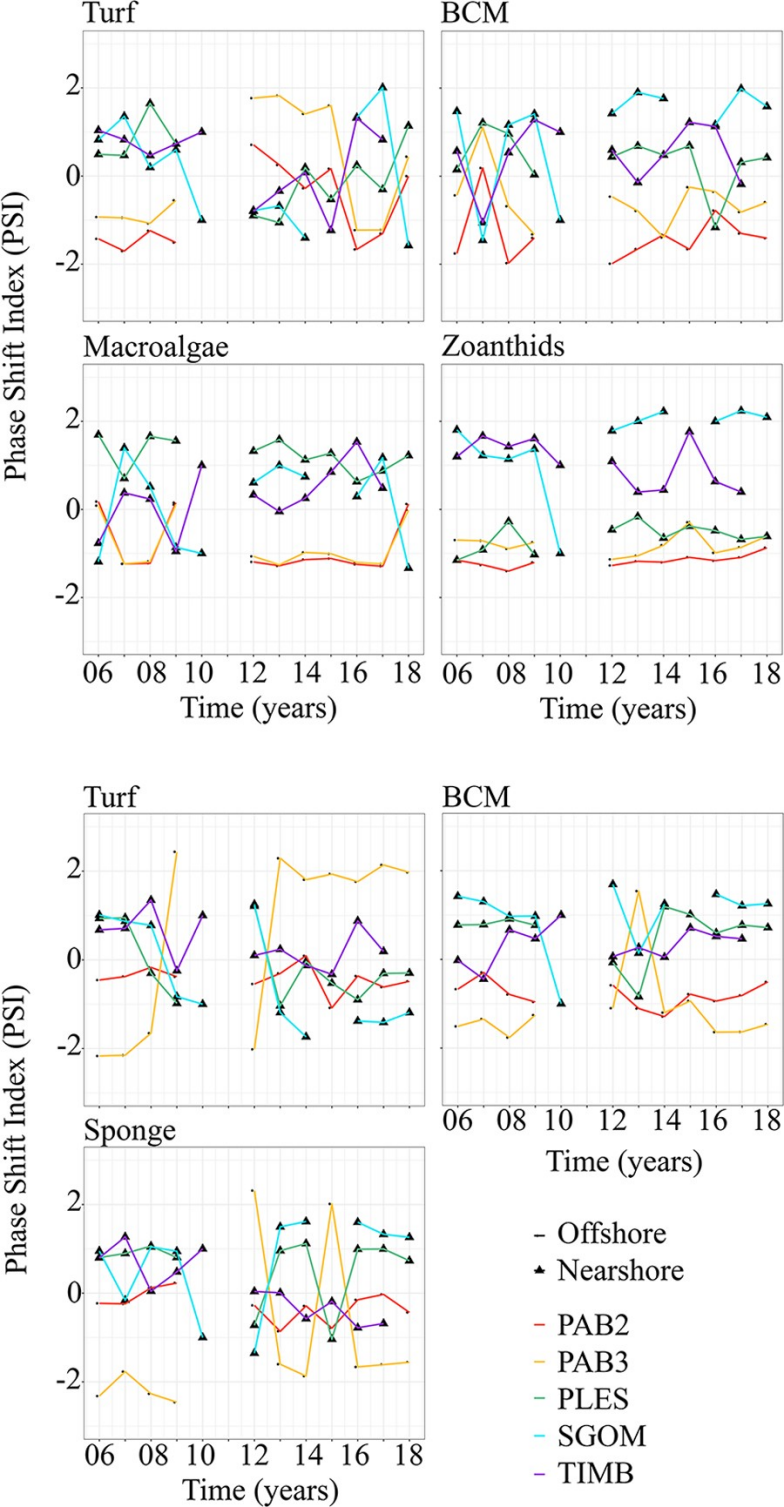
Temporal variation of the Phase Shift Index (PSI) for each major coral competitor at each site and habitat. BCM = benthic cyanobacterial mats. See Fig 1 for site codes.

For PSI comparisons using relative covers of corals *versus* BCM, macroalgae and zoanthids’ cover on pinnacles’ tops, positive values tended to be less frequent offshore (*i*.*e*., favorable to corals) than nearshore (Figs 6 and 7 and S1), indicating faster loss of space by corals in the latter. For BCM, turf and zoanthids, positive PSI values were ubiquitous on nearshore tops. Walls (Figs 6 and S1) yielded more positive values for BCM and sponges nearshore, and an opposite trend was observed offshore. Negative PSI values were more frequent for turf on offshore walls. For all coral competitors, PSI values ranged between –2.5 and + 2.5, with the smallest ranges recorded for macroalgae (Fig 6). Before 2010 and after 2016, positive PSI values were more frequent on nearshore tops for turf, while negative values were more frequent offshore, with an inverse trend between these two periods (Figs 6 and S1). Tops of SGOM presented the highest PSI values among all competitors and sites. Overall, positive PSI values were sustained on nearshore tops for macroalgae and BCM, especially after 2012 for the latter. Variability was higher between 2006 and 2009 for all sites. Except for macroalgae-dominated PLES tops, the coral-zoanthid PSI for nearshore tops were overall positive and with the highest values in SGOM. On walls, BCM and sponges sustained overall positive values nearshore, except for 2010 and 2013 (both groups), and 2015 (sponges only).

An exploratory PERMANOVA incorporating environmental variables obtained from remote sensing confirmed a strong site-level effect for both habitats, as well as a significant role of turbidity and SST, followed by smaller but yet significant effects of Instantaneous photosynthetically available radiation (iPAR) and rainfall on benthic community variation (S2 Text).

## Discussion

Habitat was the main source of community heterogeneity in the Abrolhos reefs, which sustained high coral cover over the studied decade, albeit with remarkable habitat and cross-shelf differences in benthic cover dynamics. The stability of benthic assemblages was associated with the healthy persistence of slow-growing organisms, and the high synchrony suggests that these assemblages operate under similar and potentially overwhelming drivers such as turbidity and SST (see S2 Text). However, as reef sites evolved quite distinctly along the study period, stochastic processes (e.g. chance colonization, random extinction) may also influence the dynamics of benthic cover [54, 55]. The patchy configuration of the Abrolhos’ reefs, with thousands of pinnacles surrounded by soft sediments, may indeed favor stochastic processes in benthic reef communities that dwell in relatively small areas of continuous hard bottom. Abrolhos encompasses the reefs with the highest coral cover within the SWA [39, 41], and its oddly shaped pinnacles provide contrasting light and depositional habitats that affect the local scale distribution of benthic organisms according to their intrinsic tolerances [56]. Accordingly, habitat is the primary source of variation in benthic assemblage structure [33, 39], with different abundance ranks among corals, CCA, sponges, macroalgae, turf, BCM and zoanthids. Corals are slow-growing (mm.year^-1^) and long-living (10’s to 100’s of years), and their dominance is often associated with narrow environmental fluctuation [57, 58]. However, higher coral covers were observed on nearshore walls, which is consistent with previous observations of persistent coral assemblages under turbid conditions [20]. The relatively high abundance of sponges and CCA on walls seems related to their high tolerance to low light regimes [59, 60]. Offshore, macroalgal cover was minimal, while some nearshore reefs were dominated by unpalatable forms (e.g., *Canistrocarpus*, *Lobophora*) that are weakly controlled by herbivores fishes [61, 62] due to either structural or chemical defenses. Persistent macroalgae patches may yield positive indirect effects on corals by deterring cyanobacteria that disrupt the holobiont and participate in coral tissue necrosis [63, 64]. Zoanthids occur either in small patches or as extensive carpets [65] with fast growth rates of up to 11 cm^2^.month^-1^ [66]. The dominant species in the shallow and well-illuminated pinnacles’ tops, *Palythoa caribaeorum* Duchassaing & Michelotti, 1860, is a strong competitor and important trophic link in SWA reefs [67, 68] that tolerates high turbidity and sedimentation levels [69]. However, akin to corals, *P*. *caribaeorum* seems to be vulnerable to diseases and thermal anomalies [26, 65].

Rather than coral dominance, which can be highly variable in space and independent of reef health [17, 18], stability is a more critical dimension of coral reef dynamics [7]. Stability is multidimensional and thus hard to define in a single variable but can be addressed as the composite of the coefficient of variation (CV) and community synchrony in repeated measurements [48, 49]. In Abrolhos, the identity of the dominant organism at each stratum affected how stable their assemblages were (see Figs 3 and 5). Longer-lived slower-growing organisms presented low CV and high synchrony, and such low variance is associated with the role of corals and sponges as foundation species (*sensu* [8]) in SWA reefs, providing shelter and maintaining key and non-trophic interactions with other reef organisms (e.g. [23, 70]). The association between the dynamic stability of benthic assemblages with the healthy persistence of slower-growing organisms [57, 58] seems to hold for marginal reefs that are neither dominated nor primarily built by corals [40]. However, the relatively high synchrony that we recorded suggests that the Abrolhos’ assemblages operate under similar and potentially overwhelming drivers [49, 71], such as high turbidity. This high synchrony may also be associated with the low taxonomic and functional diversity of SWA reefs, but analyses of benthic dynamics at higher taxonomic resolution are still lacking. Except for the branching *Millepora* spp., SWA coral assemblages are composed by species of either stress-tolerant or weedy life-history strategies with massive and encrusting forms, which are considered “winners” under marginal conditions [72]. Finally, it is remarkable that the relative stability of corals and sponges, when compared to shorter-lived organisms, was not related to site and habitat identity, nor their relative abundance.

Contrasting with longer-lived slow-growing organisms, assemblages of faster-growing organisms on tops had higher CV and different synchrony levels. Tops dominated by zoanthids (SGOM and TIMB) were less synchronous than those dominated by macroalgae (PLES) (Fig 5). The asynchronous dynamics of BCM-turf across sites and habitats was associated with higher and more independent fluctuations of these communities. Turfs are largely structured by macroalgae thalli [73] and form the persistent and dominant benthic matrix of the Abrolhos’ reefs, especially offshore, contrasting with ephemeral BCM that builds up episodically with negative effects on coral health [64, 74].

Phase-shifts are among the most evident symptoms of the world’s reefs decline [12, 75]. However, detection of alternate stable states is challenging because dominance may shift slowly, and baselines are often lacking [76]. Because estimates of change from repeated surveys that control for spatial heterogeneity are not widely available (but see [10]), numerous site surveys carried out along decades have been used in meta-analyzes. For instance [77], detected coral cover declines (9% per year over two decades) and macroalgae replacement in the Caribbean, but coral-to-macroalgae dominance shifts were less common and less extensive at the global scale [51]. Phase-shifts towards other fast-growing organisms have been assessed less often [13, 78]. It is also unclear whether reefs that are naturally dominated by algae (e.g. [17]) are changing toward other attractors, or if turbid zone reefs with significant coral cover are more [35] or less [36] resistant to anthropogenic stress, comprising a refuge from environmental changes [37]. Although we detected more interactions that are unfavorable to corals in nearshore reefs (e.g. positive PSI values for macroalgae and BCM on tops), the magnitude of the changes was relatively lower (PSI values of up to +2.5) than those reported by [51] (PSI values of up to +4,5), which were associated with the rapid coral cover loss across the Caribbean in the 1980’s-1990’s. In addition, our highest PSI values for turf, BCM, sponges and zoanthids indicate less frequent and intense coral-to-macroalgae shifts. Indeed, our data and evidence from previous surveys carried out five and two decades ago [39, 79] showed high algal abundance in the nearshore Abrolhos’ reefs, in comparison to offshore sites. Macroalgae may indirectly benefit Brazilian-endemic corals by reducing contacts with allelopathic cyanobacteria (e.g. [64]), and their relative high cover (up to 30% cover) should not necessarily be regarded as a phase-shift symptom [5, 17, 18], nor associated with herbivorous fish overfishing (e.g. [80]). Indeed, herbivorous fish may show strong positive feedbacks after large-scale disturbance, i.e. after mass coral bleaching and mortality [71]. While the Abrolhos’ nearshore reefs show clear signs of overfishing [81, 82], macroalgae abundance was consistently site-specific, contrasting with the prevailing paradigm that higher fish biomass alleviates coral decline [5, 80].

When compared to Caribbean reefs, before and after their 1980’s-1990’s downturn, SWA reefs exhibit lower coral diversity and cover (e.g. [83]). However, this pattern does not necessarily mean that corals and CCA were more abundant on SWA reefs for them to have been constructed, as previously assumed (e.g. [36]). Indeed, the Abrolhos reefs were built over the last 8,000 years with a relatively small participation of corals and with a major climate-induced reef growth hiatus between ~3.7 and 2.5 k years ago [40, 84], challenging the idea that long-term coral dominance (millennia to centuries) has been substituted by fast-growing organisms as a region-wide response to post-industrial stressors (e.g. [36]).

Our results contradict the expectation of higher susceptibility of SWA marginal reefs to phase shifts [36] and evidence an overall lack of specific alternative attractors, with a few exceptions. For instance, coral-zoanthid PSI values tended to increase in SGOM tops (19 to 34% increase in zoanthid cover between 2007–2017), with a negative spike during the positive SST anomaly of 2010. Despite sustaining positive values, the trend for coral-zoanthid PSI values in TIMB was the opposite (28 to 10% decrease in zoanthid cover between 2007–2017). While nearshore dominance of zoanthids seems related to higher turbidity baselines [33, 85] and positive feedback from high abundances (“abundance refuge”, *sensu* [86]), it is remarkable that their relative cover steadily increased only in SGOM, which is under the direct influence of a major dredging operation since 2002 (see S2 Text). Zoanthid dominance seemed conditioned to local chronic stress, while thermal anomalies are likely the major source of stress at the regional scale [26, 87].

Even when the drivers of coral loss are identified, their relative importance and variation in space and time are hard to disentangle. Quantifying changes in SWA reefs at meaningful temporal scales (decades) and in sites under different environmental forcing represents an irreplaceable step toward the understanding of the patterns and causes of reef degradation, which can ultimately subsidize effective management [77] and conservation planning [22, 88]. Long-term reef monitoring is a critical component of any management strategy, either locally- (e.g. fisheries, water quality) or climate-focused (e.g. emissions’ control, reforestation), but time series of benthic cover data with more than 10 years are rare and largely restricted to developed countries. The fact that we did not record a regional decline of corals toward other attractors does not mean that the Abrolhos’ reefs will keep providing key ecosystem services as thermal anomalies and terrigenous input increase [26, 87]. Contamination from the Fundão dam collapse in November 2015 was recorded in the Abrolhos reefs [89] but community-level changes may still lag several years, since sublethal contamination by heavy metals affects mostly fecundity and recruitment [90]. Indeed, the extent to which the steadily increasing land-based stressors undermine coral cover in SWA turbid zone reefs is hitherto unclear. Remarkably though, slow systems live on borrowed time during protracted transient periods driven by local stressors [76]. Therefore, it is still worth restraining the stressors that clearly contributed to coral reef decline in the last decade, such as sedimentation from dredging discharges near coral reefs. Preventing new alternate states from fully eventuating is critical to prevent a regional-level collapse of coral reefs and the livelihoods of billions of people that depend on healthy coastal ecosystems.

## Supporting information

S1 Text Position and dispersion effects between and within habitats. (DOCX)

S2 Text Abrolhos reefs, Brazil: Historical stressors, management regimes and potential drivers of benthic assemblage dynamics. (DOCX)

S1 Fig PSI frequency. Frequency of Phase Shift Index (PSI) values for each main coral competitor at both arcs and habitats. BCM = benthic cyanobacteria mats.
(TIF)

S1 Table Benthic cover across years. Percent cover of the different groups for each year, site, and habitat sampled in the Abrolhos reefs between 2006 and 2018.
(DOCX)
