# Supplementary material for: Decadal (2006-2018) dynamics of Southwestern Atlantic’s largest turbid zone reefs
Source: PLoS One. 2021 Feb 22;16(2):e0247111. doi: 10.1371/journal.pone.0247111 (PMC7899327; doi:10.1371/journal.pone.0247111)
Supplement: S1 Text — (DOCX) [file pone.0247111.s001.docx]

**S1 Text.  Position and dispersion effects between and within habitats.**

Due to the odd morphology of the Abrolhos’ pinnacles, with steep shaded walls and flat well-lit tops (Figure A), previous work suggested that habitat (tops and walls) is the main source of variation in the region’s benthic assemblages (e.g. [1, 2]). Here, we further developed this assumption by performing a Permutational Multivariate Analysis of Variance (PERMANOVA) and a Permutational Analysis of Multivariate Dispersions (PERMDISP) using yearly means for each habitat and site combination [3,4]. These results (table and Figure B) show a large amount of variation associated to habitat, and we concluded that both habitats should be treated separately in order to assess other sources of spatial and temporal variation in the benthic assemblages.


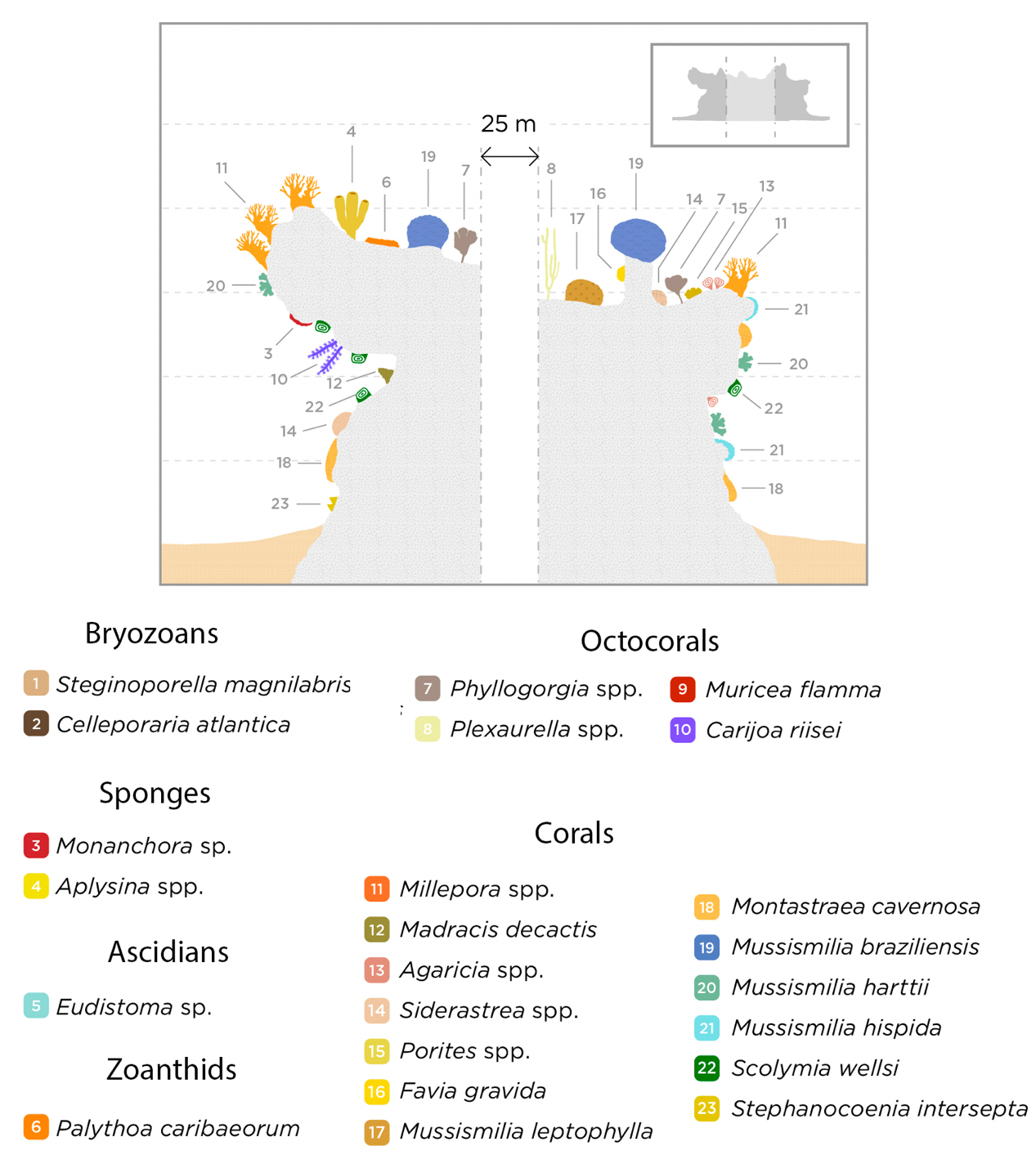


Figure A. Schematic representation of a typical reef pinnacle in the Abrolhos Reefs, with the preferred habitat of selected benthic species. Figure drawn by the authors.

**Table.** Summary of the results of the Permutational multivariate analysis of variance (PERMANOVA) and Permutational analysis of multivariate dispersions (PERMDISP). Significant values (p ≤ 0.05) marked with an asterisk. Df = Degrees of freedom; SS = Sum of Squares.

|  | Df | SS | MS | F | R2 | Pr(>F) |
| --- | --- | --- | --- | --- | --- | --- |
| **PERMANOVA** | |  |  |  |  |  |
| Habitat | 1 | 1548.6 | 1548.55 | 67.927 | 0.26855 | ***** |
| Site | 4 | 1006.9 | 251.72 | 11.042 | 0.17461 | ***** |
| Habitat:Site | 4 | 931.2 | 232.81 | 10.212 | 0.16149 | ***** |
| Residuals | 100 | 2279.7 | 22.80 |  | 0.39535 |  |
| Total | 109 | 5766.4 |  |  | 1.00000 |  |
| **PERMDISP** |  |  |  |  |  |  |
| Habitat | 1 | 1.67 | 1.6698 | 0.2969 |  | 0.587 |
| Residuals | 108 | 607.39 | 5.6240 |  |  |  |


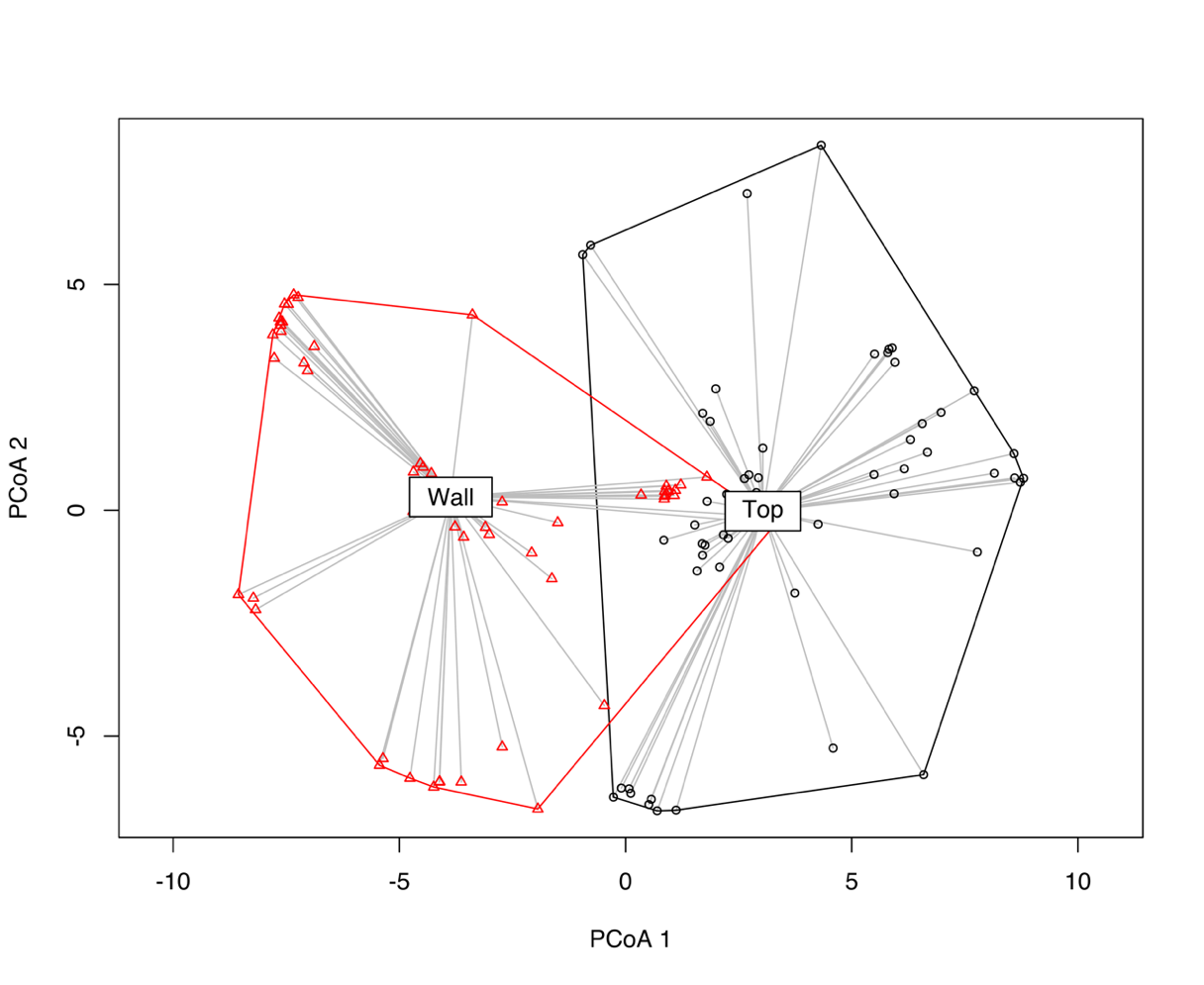


**Figure B.** Principal coordinates analysis (PCoA) plot of Permutational analysis of multivariate dispersions (PERMDISP). Red triangles = Wall, Black circles = Top.

**References**

1. Francini-Filho RB, Coni EOC, Meirelles PM, Amado-Filho GM, Thompson FL, Pereira-Filho GH, et al. Dynamics of coral reef benthic assemblages of the Abrolhos Bank, Eastern Brazil: inferences on natural and anthropogenic drivers. PloS One. 2013; 8(1): e54260.
2. Bastos AC, Moura RL, Moraes FC, Vieira LS, Braga JC, Ramalho LV, et al. Bryozoans are major modern builders of South Atlantic oddly shaped reefs. Sci Rep. 2018; 8: 9638.
3. Anderson MJ. A new method for non‐parametric multivariate analysis of variance. Austral Ecology 2001; 26: 32 - 46.
4. Anderson MJ, Walsh DC. PERMANOVA, ANOSIM, and the Mantel test in the face of heterogeneous dispersions: what null hypothesis are you testing? Ecological Monographs 2013; 83: 557-574.
