# Supplementary material for: Decadal (2006-2018) dynamics of Southwestern Atlantic’s largest turbid zone reefs
Source: PLoS One. 2021 Feb 22;16(2):e0247111. doi: 10.1371/journal.pone.0247111 (PMC7899327; doi:10.1371/journal.pone.0247111)
Supplement: S2 Text — (DOCX) [file pone.0247111.s002.docx]

**S2 Text. Abrolhos Reefs, Brazil: Historical stressors, management regimes and potential drivers of benthic assemblage dynamics.**

Here we present an overview of the main climatic and anthropogenic stressors [1] that operate over the Abrolhos' reefs (formerly reviewed by [2-3]) and explore the potential drivers of benthic assemblage dynamics between 2006 and 2018. .

Abrolhos represents Southwestern Atlantic's largest, richest, and best-protected reefs, but the region is under intense land-use changes, population increase and overfishing. Industrialization is steadily growing, including a close interaction with the world's largest iron ore mining and wood pulp companies, the second and third main commodities exported by Brazil, respectively. Albeit sparse, we included the more scattered information from the onset of Portuguese colonization (XVI Century) to the early XXI Century.

**Thermal stress and coral bleaching:** All mass coral bleaching events recorded in Abrolhos coincided with positive phases of the El Niño Southern Oscillation (ENSO) (Fig A) in 1993, 1998 (First Global Bleaching Event), 2003, 2010, 2016-2017 [4]. There is no mention to the phenomenon in older accounts (e.g. [5-6]). The main positive thermal anomalies recorded during our study (2006-2018) reached 10.60 Degree Heating Week (DHW, see [7]) in 2010 and 10.95 DHW in 2016, coinciding with the Second and Third Global Bleaching Events, respectively (Fig B). Information about coral bleaching prevalence and mortality in 1993, 1998, 2003 and 2010 is scarce (see [4]). During the 2016-2017 mass bleaching event, 33 and 25% of the colonies bleached on nearshore reefs’ tops and walls, respectively, and 37 and 27% bleached on offshore reefs’ tops and walls, respectively. Overall mortality reached ~2% of coral colonies. Despite the aforementioned relatively low coral mortality (but see [8]), it is plausible to assume that the recent and more severe bleaching episodes influenced community dynamics.

**Turbidity**: The Abrolhos reefs are among the world's most turbid living reefs [9]. Turbidity and sedimentation presents marked seasonality, cross-shelf and longshore gradients, which are associated with continental sourcing, transport and winter-storm resuspension [10]. Between 2006 and 2019, turbidity derived from remote sensing [light attenuation coefficient at 490 nm (Kd490)] was minimum on offshore reefs (Parcel dos Abrolhos, 0.076 m-1 yearly average) and in the northernmost coastal reef (Timbebas, 0.079 m-1), and maximum on the other coastal reefs (Sebastião Gomes and Pedra de Leste, 0.151 and 0.162 m-1, respectively) (Fig B).


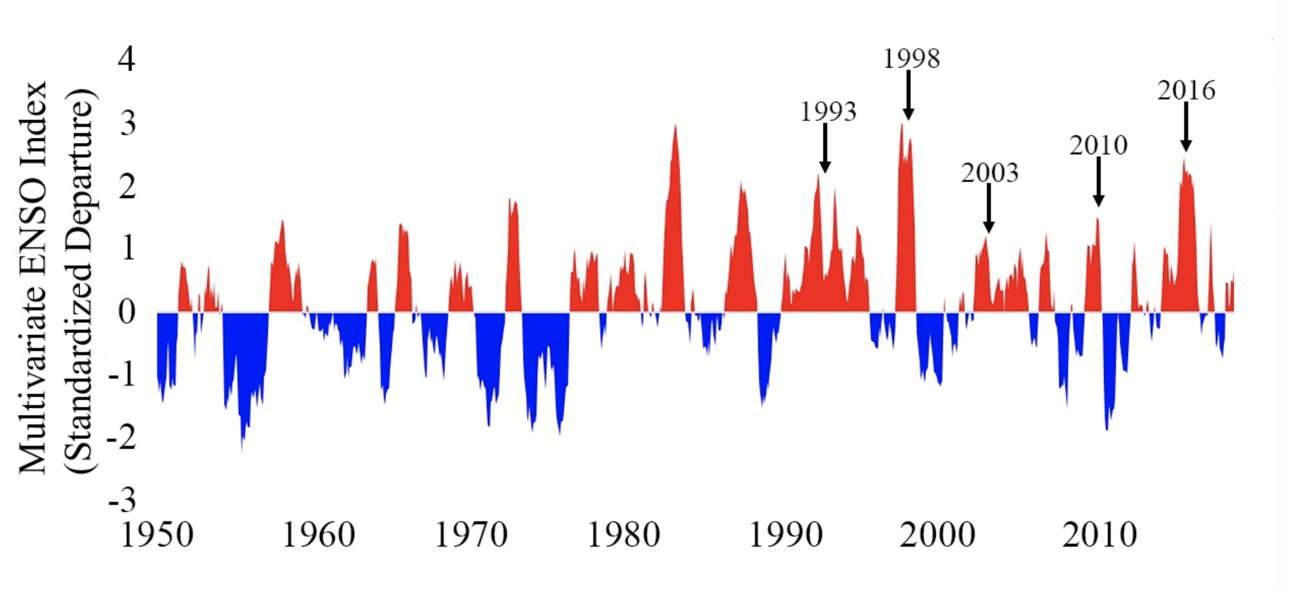


**Figure A.** Multivariate ENSO Index [11] between 1950 and 2019. Values were obtained from NOAA’s Earth System Research Laboratory (esrl.noaa.gov/psd/enso/mei) in April 07, 2020. Black arrows indicate mass coral bleaching events recorded in the Abrolhos Reefs.


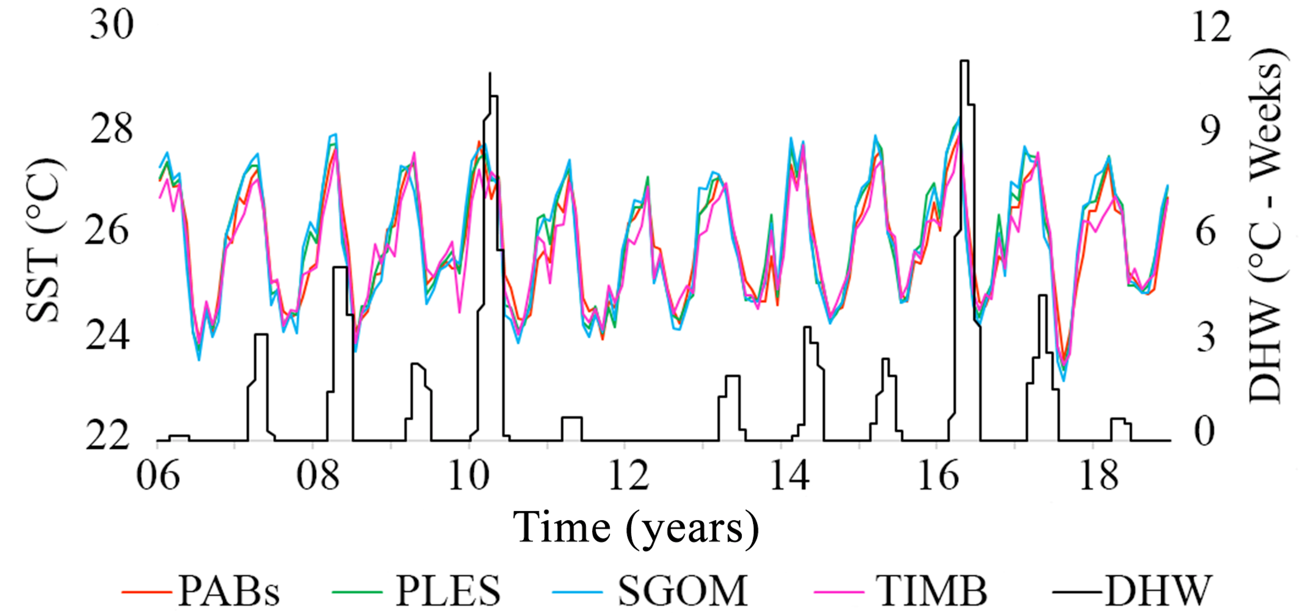


**Figure B.** Sea surface temperatures (SST) in the five study sites between 2006 and 2019 (colored lines). The black line represents the Degree Heating Week index [7], obtained from NOAA’s Coral Reef Watch (coralreefwatch.noaa.gov).


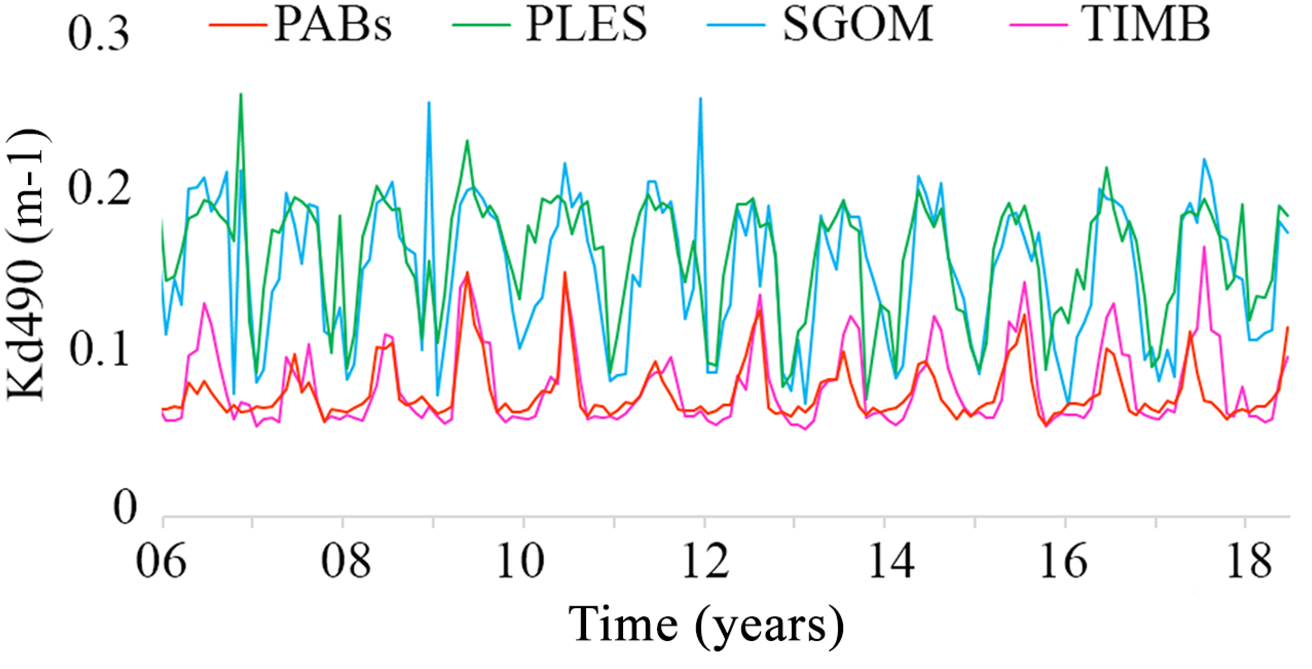


**Figure C.** Variation in the light attenuation coefficient at 490 nm (Kd490) between 2006 and 2019 in offshore (PABs) and coastal (PLES, SGOM and TIMB) reef sites. See main text for site codes. Data obtained from NOAA’s Easier Access to Scientific Data database (coastwatch.pfeg.noaa.gov/erddap).

**Coral diseases:**A white plague-like coral disease was first recorded in 2005 and became widespread from 2006 on [12]. Its prevalence reached 12.5% of the colonies and increased during summers. At least five other coral disease syndromes are recorded in the region [12], all associated to thermal anomalies and microbiome disruptions due to water quality loss. Despite the relatively large amount of information about coral diseases in Abrolhos (e.g. [13-14]), there is no specific systematic monitoring since 2008.

**Dams and land use:** During the 16^th^ and 17^th^ centuries, wood extraction and sugar cane plantations near the coast were Brazil’s main economic activity [15]. From 1700 to 1750, gold and diamonds’ mining led to intense deforestation in the upper reaches of the Jequitinhonha and Doce watersheds (Minas Gerais State), with a regional population increase from 30,000 to 407,000 inhabitants [16]. The so-called Gold Cycle was followed by large-scale crops and pastures [17-18]. The degradation of the mid and lower reaches of the Doce river escalated more recently due to iron ore mining, which started in the 1950’s (largely by Vale, the world’s largest iron ore producer) and is associated with intense urbanization. The Bahia-Minas Railroad (active between 1882 and 1966) was the main deforestation axis in the region adjacent to the Abrolhos reefs, which escalated between the 1940's and 1970's (Fig C). Road infrastructure further expanded deforestation, especially after the conclusion of BR-101 in 1960. Currently, the Atlantic Rainforest is highly fragmented and retains less than 7% of its original cover (Fig C), with eucalyptus, cattle ranching, and sugarcane as its main substitutes [17-18].

The Abrolhos’ reefs are bounded by the Jequitinhonha and Doce watersheds [19-20], which drain a yearly average of 409 and 900 m^3^.s^-1^, respectively. These basins are currently responsible for about 7% of Brazil's hydropower energy generation, with dams established in 1973 (Mascarenhas), 2004 (Risoleta Neves), 2005 (Aimorés) and 2009 (Baguari) in the main course of the Doce river, and in 2003 (Itapebí) and 2006 (Irapé) in the Jequitinhonha river (Fig D). Smaller coastal watersheds adjacent to the Abrolhos reefs contribute with a yearly average flow of 142 m^3^.s^-1^ (Fig D, insert)


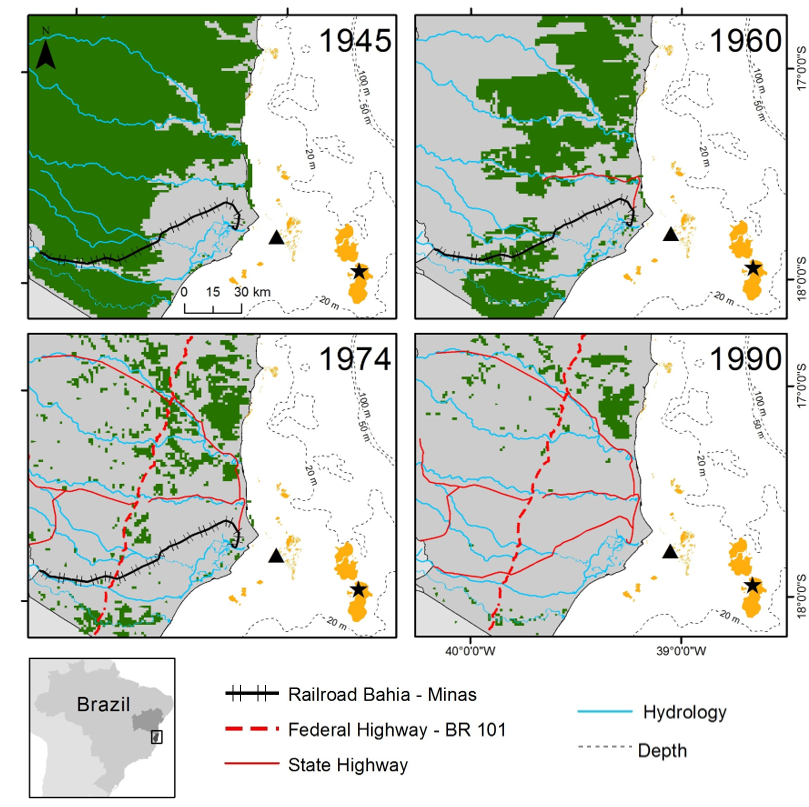


**Figure C.** Pictorial timeline of deforestation, railroad and road building in the region adjacent to the Abrolhos’ reefs. Black triangle represents the location of the Pedra de Leste (PLES) inshore reef site and the black star represents offshore reef site PAB2 in the Parcel dos Abrolhos. Maps drawn by the authors using ArcGIS 10.6 (www.esri.com) and based on geospatial raster data freely available from the Marinha do Brasil (www.marinha.mil.br/dhn/), as well as data from [21] and the New York Botanical Garden (www.nybg.org/bsci/res/bahia/Defor.html).


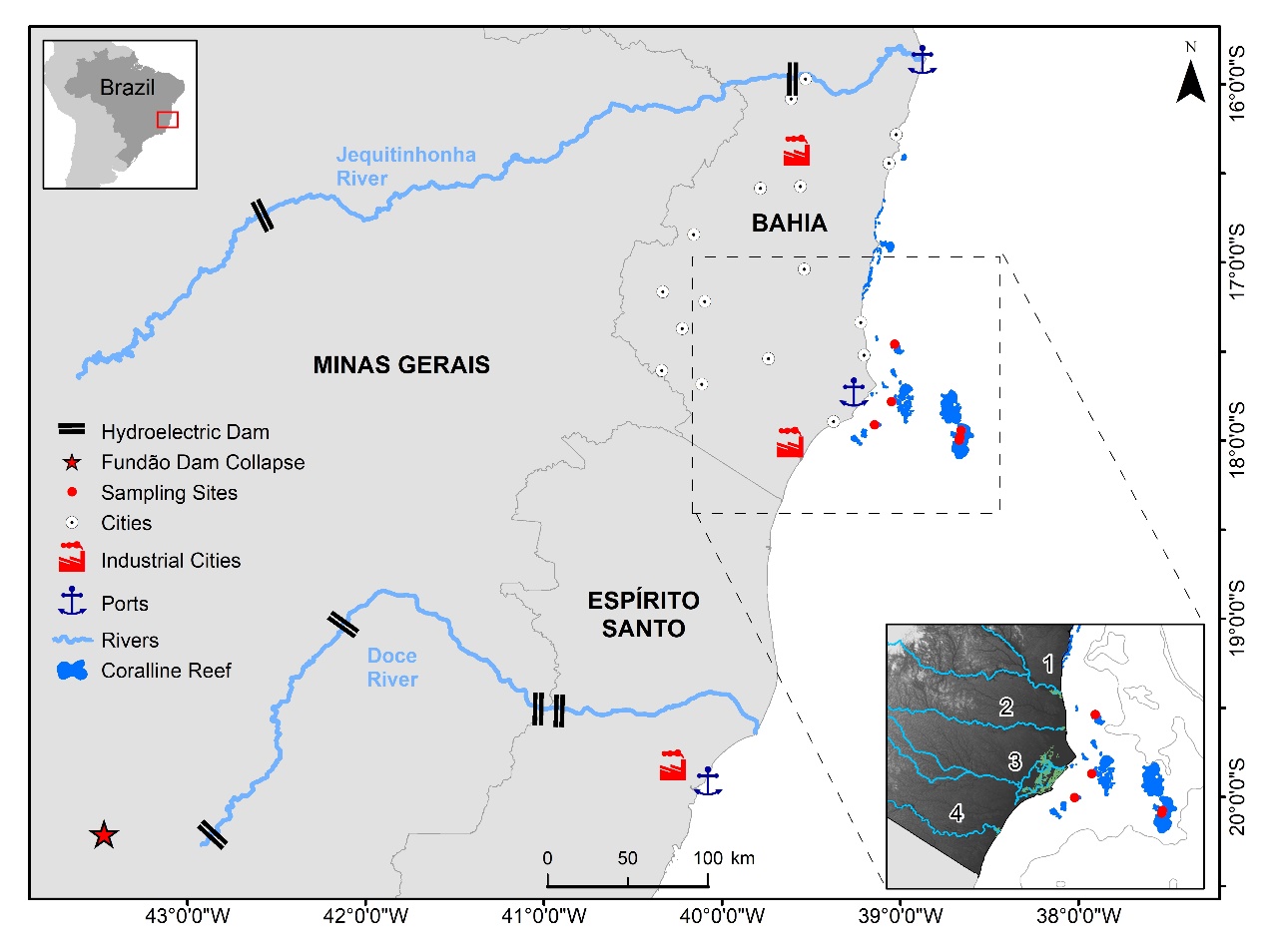


**Figure D.** The two main hydrographic basins that bound the Abrolhos reefs. Cities in Bahia state (circles) correspond to the municipalities used in the population growth compilation. Hydropower dams, wood pulp processing plants and their associated ports are also depicted. The red star represents the site of the Fundão Dam collapse. The insert shows the local drainages near the reefs: 1) Jucuruçu river, 2) Itanhém river, 3) Peruipe river, 4) Mucuri river. Gray line corresponds to the shelf break at 100 m depth. Coral reef sampling sites are shown as red dots. Map drawn by the authors using ArcGIS 10.6 (www.esri.com) and based on geospatial data freely available from the Instituto Brasileiro de Geografia e Estatística (<www.ibge.gov.br>) and Marinha do Brasil (www.marinha.mil.br/dhn/).

**Urbanization and industrialization:** Current population in the Doce and Jequitinhonha watersheds (Minas Gerais, Espírito Santo, and Bahia states) surpasses 4 million inhabitants. Southern Bahia alone has 21 municipalities at less than 100 km from the coastline, with a population growth of 52%, from 416 to 874 thousand inhabitants between 1970 and 2019. This trend is associated to the industrialization of municipalities with wood pulp industry infrastructure (e.g. Teixeira de Freitas, Eunápolis, Mucuri) and/or mass tourism (Porto Seguro) (Fig E). Sewage treatment is lacking from all municipalities and the Human Development Index varies from mid (Teixeira de Freitas: 0.685) to low (Vereda: 0.557). Large scale land acquisition by the wood pulp industry started in the mid 1980's, accelerated in the early 1990's, and pressed urbanization of some mid-sized cities (Fig E). Wood pulp processing plants in Mucuri and Eunápolis started to operate in 1992 and 2005, respectively. Oil and gas exploration tends to increase regionwide [22]. A gas rig (Peroá-Cangoá) operates since 2006, 50 km to the south of Sebastião Gomes reef (sampling site SGOM).

**Dredging and sedimentation:** A relatively large dredging operation occurs in the mouth of the Caravelas estuary, in order to facilitate the traffic of barges that carry eucalyptus logs for Fibria (world’s largest cellulose company). This operation started in 2002 by removing 887,000 m^3^ of sediments and was followed by a yearly average dredging volume of 250,000 m^3^. Dredging occurs during summers, supposedly to reduce sediment dispersion, but sediment discharge takes place about 12 km from the Sebastião Gomes reef, one of the sites monitored during our study (Fig F). Dredging operations near the reefs have been a constant source of conflicts between the third-party dredging companies and artisanal fishers [23]. Despite several sedimentation studies [24-27], the effects from dredging over nearshore reefs have not been previously assessed.


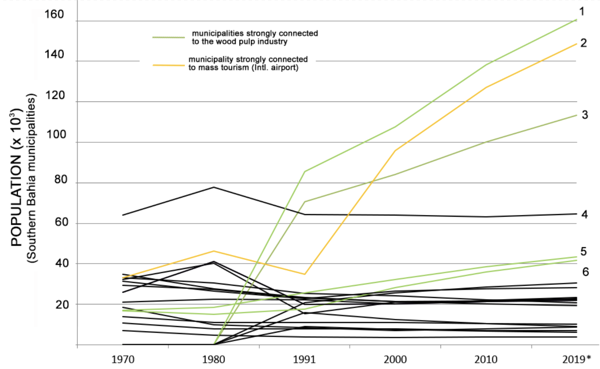


**Figure E.** Human population trends in the 21 coastal municipalities of Southern Bahia (location in Fig C) showing the association with the wood pulp industry (green lines) and mass tourism (yellow line). Municipalities: 1=Teixeira de Freitas (machinery commerce and inputs for eucalyptus plantations); 2=Porto Seguro (mass tourism, international airport); 3=Eunápolis (wood pulp processing plant); 4=Itamaraju; 5=Nova Viçosa (dormitory city for industrial workers in Mucuri and tourism); 6=Mucuri (wood pulp processing plant). Itamaraju lacks industrial infrastructure and was the only municipality with >50,000 inhabitants with a relatively stable population. Source: Instituto Brasileiro de Geografia e Estatística (www.ibge.gov.br).


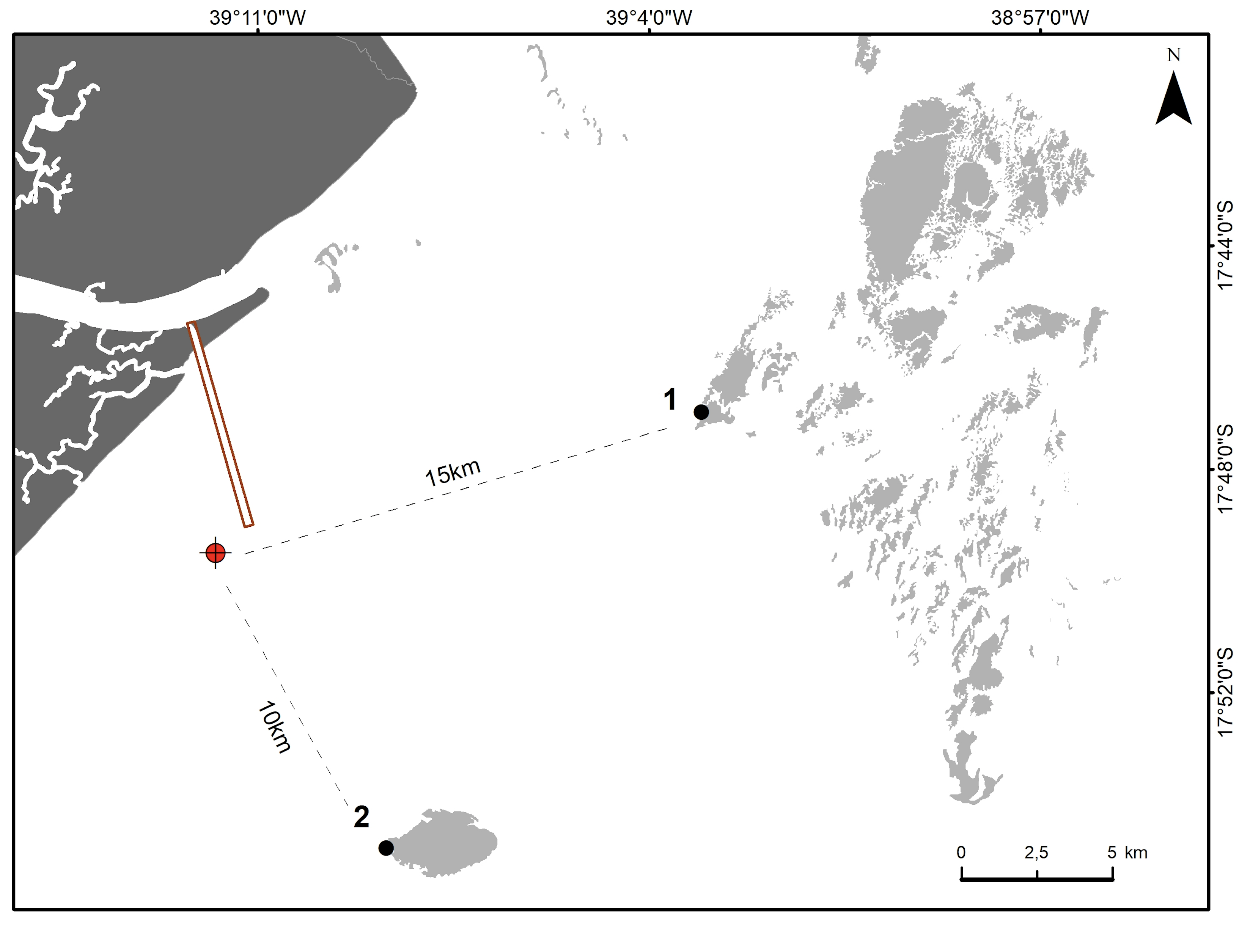

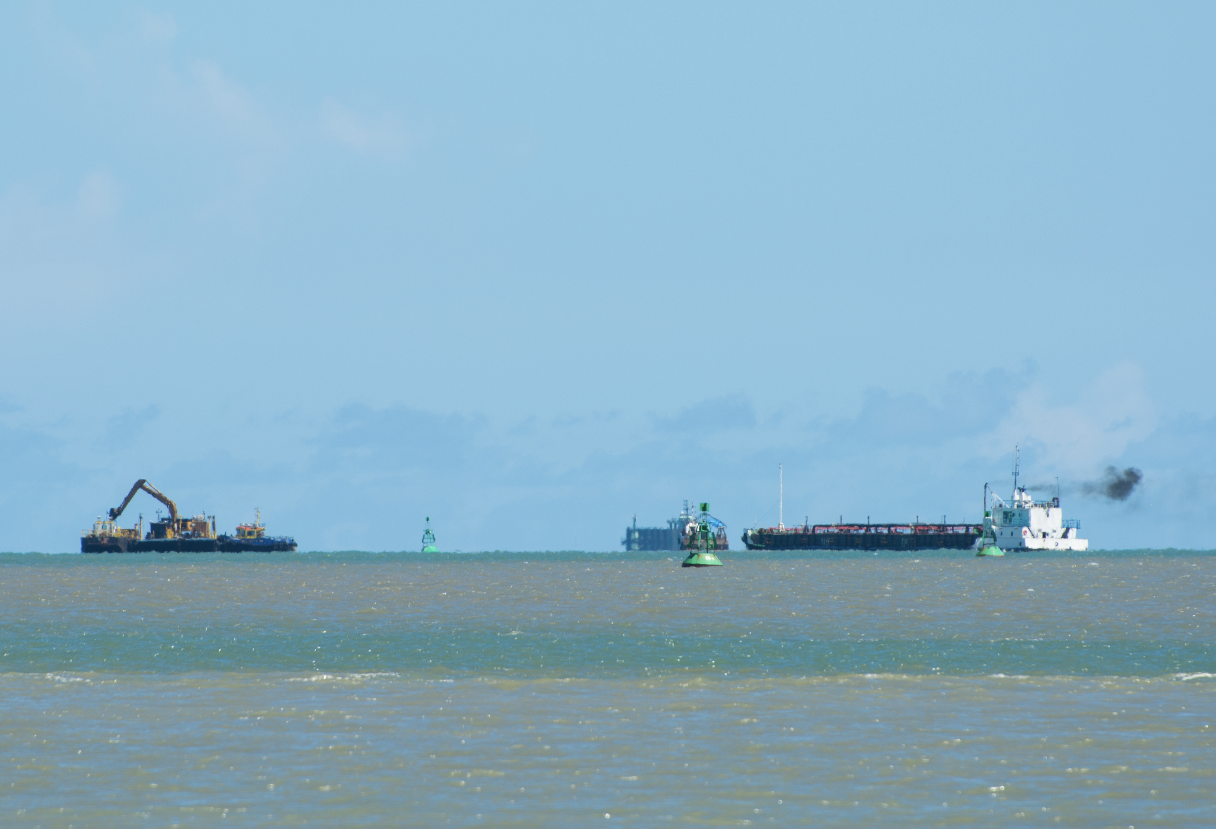


**Figure F.** Map showing the location of the dredged channel (red line) and disposal site (red circle) near the sampling sites Pedra de Leste (PLES, 1) and Sebastião Gomes reef (SGOM, 2). The lower photograph shows two dredges and an eucalyptus barge in the middle. Photo by R. L. Moura. Map drawn by the authors using ArcGIS 10.6 (www.esri.com) and based on geospatial data freely available from Marinha do Brasil (www.marinha.mil.br/dhn/).

**Mining disaster:**In November 2015 the Fundão mining tailings’ dam collapsed (see location in Fig C) and severely affected the mid and low reach of the Doce river [27], and also ensued a turbidity plume with at least 7,000 km^2^ in the ocean [28-29]. Contaminants reached the southernmost reefs of the Abrolhos Shelf [30-31], and it is possible that all coastal reefs are currently under chronic input of dissolved heavy metals and other toxic materials [32-33]. Detectable effects over benthic communities may occur in the long term because effects of contaminants may impair the reproductive output of corals [34]. A systematic assessment of the environmental impact over the reefs began only two years after the disaster.

**Fisheries and marine management:** Grouper fishing and export of salted fish is recorded as one of the main economic activity in the Abrolhos' region in the XVI Century [35]. Extraction of olivid gastropods, which were confounded with cypraeid cowries (=cauri) and used as currency in the slave trade, was also an important XVII Century extractive activity in Caravelas [36]. Whaling was important in the late XIX and early XX Century, with a relatively large (30-50 whales.year^-1^) pre-industrial whaling station in this same town operating between 1847 and 1929 [37]. Artisanal fisheries with hook-and-line and sail boats without engines predominated till the 1960’s, when diesel engines and nylon were introduced into the local fisheries. Shrimp trawling near reefs and spearfishing were introduced in the 1970’s and 1980’s, respectively, and fisheries are currently the main activity for more than 20,000 people across the region [3,35,38]. There are several examples of overfished stocks (e.g. [39]), including large herbivores [35].

Fisheries management includes a few rules with virtually no enforcement [35]. Exceptions include snook and shrimp trawling seasons, as well as Marine Protected Areas that include multiple-use community co-management in the Cassurubá Extractive Reserve (largely estuarine, established in 2009) and in the Corumbau Marine Extractive reserve (established in 2000), and no-take zoning in the offshore reefs within the Abrolhos National Marine Park (ANMP), established in 1983 and under federal jurisdiction. The ANMP is discontinuous and include the nearshore Timbebas reef – TIMB, which represents a poorly enforced no-take portion of the park [3,40]. The other nearshore reefs included in our sampling, Sebastião Gomes(SGOM) and Pedra de Leste (PLES) are within a paper park declared in 1993 and under the Bahia State jurisdiction (Ponta da Baleia/Abrolhos Environmental Protection Area).

**Drivers of benthic assemblage dynamics**

The potential drivers of change in benthic assemblages were explored with PERMANOVA using Euclidian distances among log-ratio transformed relative abundances of all benthic categories [41,42]. We used a set of variables (annual means) that are usually associated with changes in benthic assemblages and were available from remote sensing between 2003 and 2018. Nighttime sea surface temperatures (SST) were obtained from the Physical Oceanography Distributed Active Archive Center (podaac.jpl.nasa.gov). Instantaneous photosynthetically available radiation (iPAR) and Kd490 were acquired from NOAA’s Easier Access to Scientific Data database (coastwatch.pfeg.noaa.gov/erddap). Parameters K490, iPAR and SST are products of the MODIS sensor. Ultraviolet data (DNA-damage UV dose) was obtained from the Scanning Imaging Absorption Spectrometer for Chartography Atmospheric (SCIAMACHY) available at the Tropospheric Emission Monitoring Internet Service - TEMIS (http://www.temis.nl/index.php). Rainfall data was obtained from station 83498 of Brazil’s National Meteorology Institute – INMET (https://portal.inmet.gov.br). Spatial resolution of data was 4 km for all variables, with the exception of UV (27 km). Data integration and geostatistics were carried out in ArcGIS 10.6.

For pinnacles' tops, a strong site effect was detected (R^2^=0.44; p<0.00001), overcasting the contribution of other variables for the overall explained variation in assemblage structure (Table). Site identity was a major source of variation among assemblages. Intra-group dispersion among sampling units did not vary significantly among sites, as demonstrated by a test for homogeneity of multivariate dispersions (PERMDISP F = 0.43; p = 0.79). However, when site was excluded and temporal interactions were considered, the overall variance associated with the environmental variables was even higher (R^2^ = 0.62) (Table). These results comprise further evidence for strong site-level effect for both habitats, as well as a significant role of turbidity and SST, followed by smaller but yet significant effects of iPAR and rainfall on benthic community variation. The lack of significant interaction between site identity and environmental variables suggests that their effect is regional.

Assemblages changed significantly over the years (R^2^=0.20; p=0.005) and the influence of the explanatory variables had a significant interaction with time. The inclusion of an interaction term with time improved the overall explained variance. Sea surface temperature (SST) and turbidity (Kd490) were the most influential variables. While turbidity was the most influential driver, the high contribution of SST and UV and iPAR, and especially their interaction with time (R^2^ = 0.16, p = 0.02), seems to be related with the anomalous high temperatures during heatwaves (see Teixeira et al. [4]).

For walls, intra-group dispersion (PERMDISP, F=5.21; p = 0.001) was higher than that observed for tops. Still, site identity remained as an important source of variation among assemblages. Akin to tops, the inclusion of site identity within the PERMANOVA overcasted the effect of other variables. Again, interaction between environmental variables and time was significant and improved the total explained variance (R^2^). Turbidity and SST remained as the most influential variables.

Table. Summary of Permutational Analyses of Variance (PERMANOVA) exploring the effect of environmental variables over benthic assemblage structure (tops and walls). *=0.01; **0.001; ***<0.001

|  | Df | SS | Mean Square | F | R^2^ | Pr (>F) |
| --- | --- | --- | --- | --- | --- | --- |
| Pinnacle's Tops |  |  |  |  |  |  |
| Kd490 | 1 | 340.81 | 340.81 | 19.33 | 0.16 | *** |
| SST | 1 | 90.55 | 90.55 | 5.14 | 0.04 | ** |
| iPAR | 1 | 63.03 | 63.03 | 3.58 | 0.03 | * |
| UV | 1 | 82.29 | 82.29 | 4.67 | 0.04 | ** |
| Rainfall | 1 | 89.17 | 89.17 | 5.06 | 0.04 | ** |
| Year | 10 | 422.20 | 42.22 | 2.39 | 0.2 | ** |
| Kd490:Year | 11 | 323.71 | 29.43 | 1.67 | 0.15 | . |
| SST:Year | 10 | 339.55 | 33.95 | 1.93 | 0.16 | * |
| iPAR:Year | 7 | 173.55 | 24.79 | 1.41 | 0.08 | 0.16 |
| Residuals | 11 | 193.94 | 17.63 |  | 0.09 |  |
| Total | 54 | 2118.81 |  |  | 1.00 |  |
| Pinnacle's Walls |  |  |  |  |  |  |
| Kd490 | 1 | 222.80 | 222.80 | 14.75 | 0.11 | *** |
| SST | 1 | 92.38 | 92.38 | 6.12 | 0.04 | *** |
| iPAR | 1 | 42.60 | 42.60 | 2.82 | 0.02 | * |
| UV | 1 | 68.57 | 68.57 | 4.54 | 0.03 | ** |
| Rainfall | 1 | 16.47 | 16.47 | 1.09 | 0.01 | 0.35 |
| Year | 10 | 656.41 | 65.64 | 4.35 | 0.31 | *** |
| Kd490:Year | 11 | 260.31 | 23.66 | 1.57 | 0.12 | . |
| SST:Year | 10 | 364.57 | 36.46 | 2.41 | 0.17 | ** |
| iPAR:Year | 7 | 208.85 | 29.84 | 1.98 | 0.1 | * |
| Residuals | 11 | 166.12 | 15.10 |  | 0.08 |  |
| Total | 54 | 2099.07 |  |  | 1.00 |  |

**References:**

1. Borics G, Várbíró G, Padisák J. Disturbance and stress: different meanings in ecological dynamics? Hydrobiologia. 2013; 711:1-7. doi: 10.1007/s10750-013-1478-9.
2. Leão ZMAN, Kikuchi RKP. A relic coral fauna threatened by global changes and human activities, Eastern Brazil. Mar Pollut Bull. 2005; 51: 599-611. doi: 10.1016/j.marpolbul.2005.04.024.
3. Moura RL, Secchin NA, Amado-Filho GM, Francini-Filho RB, Freitas MO, Minte-Vera CV, et al. Spatial patterns of benthic megahabitats and conservation planning in the Abrolhos Bank. Cont Shelf Res. 2013; 70: 109-117. doi: 10.1016/j.csr.2013.04.036.
4. Teixeira CD, Leitão RLL, Ribeiro FV, Moraes FC, Neves LM, Bastos AC, et al. Sustained mass coral bleaching (2016-2017) in Brazilian turbid-zone reefs: taxonomic, cross-shelf and habitat-related trends. Coral Reefs. 2019; 38: 801-813. doi: 10.1007/s00338-019-01789-6.
5. Hartt CF. Geology and physical geography of Brazil. Boston: Boston, Fields, Osgood and Co; 1870.
6. Laborel J. Les peuplements de Madréporaires des côtes tropicales du Brésil. Ann Univ Abidjan, Ser E. 1969 ; 2: 1-261.
7. Liu G, Heron SF, Eakin CM, Muller-Karger FE, Vega-Rodriguez M, Guild LS, et al. Reef-scale thermal stress monitoring of coral ecosystems: New 5-km global products from NOAA Coral Reef Watch. Remote Sens. 2014; 6:11579–11606. doi: 10.3390/rs61111579.
8. Duarte GAS, Villela HDM, Deocleciano M, Silva D, Barno A, Cardoso PM, et al. Heat waves are a major threat to turbid coral reefs in Brazil. Front Mar Sci. 2020; 7: 179. doi: 10.3389/fmars.2020.00179.
9. Leão ZMAN, Ginsburg RN. Living reefs surrounded by siliciclastics sediments: the Abrolhos coastal reefs, Bahia, Brazil. Proc 8^th^ Coral Reef Sym. 1997; 2: 1767-1772.
10. Segal B, Evangelista H, Kampel M, Gonçalves AC, Polito OS, Santos EA. Potential impacts of polar fronts on sedimentation process at Abrolhos coral reef (Southwest Atlantic Ocean/Brazil). Cont Shelf Res. 2008; 28: 533-544. doi: 10.1016/j.csr.2007.11.003.
11. Wolter K, Timlin MS. Monitoring ENSO in COADS with a Seasonally Adjusted Principal Component Index. Proceedings of the 17th Climate Diagnostics Workshop, Norman. 1993; 52-57.
12. Francini-Filho RB, Moura RL, Thompson FL, Reis RM, Kaufman L, Kikuchi RKP, et al. Diseases leading to accelerated decline of reef corals in the largest South Atlantic reef complex (Abrolhos Bank, eastern Brazil). Mar Pollut Bull. 2008; 56: 1008-1014. doi: 10.1016/j.marpolbul.2008.02.013.
13. Francini-Filho RB, Reis R, Meirelles P, Moura R, Thompson F, Kikuchi R, et al. Seasonal prevalence of white plague like disease on the endemic Brazilian reef coral *Mussismilia braziliensis*. Lat Am J Aquat Res. 2010; 38(2): 292-296. doi: 10.3856/vol38-issue2-fulltext-16. doi: 10.3856/vol38-issue2-fulltext-16.
14. Alves-Junior N, Maia-Neto OS, Silva BSO, Moura RL, Francini-Filho RB, Castro CB, et al. Diversity and pathogenic potential of vibrios isolated from Abrolhos Bank corals. Environ Microbiol Rep. 2010; 2(1): 90-95. doi: 10.1111/j.1758-2229.2009.00101.x.
15. Burns EB. A history of Brazil. New York: Columbia University Press; 1980.
16. Dean W. A ferro e fogo: a história e a devastação da mata atlântica brasileira. São Paulo: Cia das Letras; 1996.
17. Espindola HS. Sertão, território e fronteira: expansão territorial de Minas Gerais na direção do litoral. Fronteiras. 2008; 10(17): 69-96.
18. Sobreira F. Mineração do ouro no período colonial: alterações paisagísticas antrópicas na serra de Ouro Preto, Minas Gerais. Quaternary and Environmental Geosciences. 2014; 5(1): 55-65.
19. Coelho ALN. Bacia hidrográfica do Rio Doce (MG/ES): uma análise socioambiental integrada. Geografares. 2009; 7: 131-145.
20. Sonter LJ, Barrett DJ, Moran C, Soares-Filho B. Mining, deforestation and conservation opportunities: A case study of the Quadrilátero Ferrífero land use change dynamics. In XVI Brazilian Remote Sensing Symposium (SBSR). Foz do Iguaçu, Brazil. 2013.
21. Mendonça JR, Carvalho AM, Silva LA, Thomas WW. 45 Anos de desmatamento no Sul da Bahia, remanescentes da Mata Atlântica - 1945, 1960, 1974, 1990. Projeto Mata Atlântica Nordeste, CEPEC, Ilhéus, Bahia, Brazil. 1994.
22. Marchioro GB, Nunes MA, Dutra GF, Moura RL, Pereira PGP. Avaliação dos impactos da exploração e produção de hidrocarbonetos no Banco dos Abrolhos e adjacências. Megadiversidade. 2005; 1(2): 225-310.
23. Previero M, Gasalla MA. Risk assessment of small-scale reef fisheries off the Abrolhos Bank: snappers and groupers under a multidimensional evaluation. Fish Manag Ecol. 2019; 00: 1-17. doi: 10.1111/fme.12406.
24. Dutra LXC, Kikuchi RKP, Leão ZMAN. Effects of sediment accumulation on reef corals from Abrolhos, Bahia, Brazil. J Coast Res. 2006; 39: 633-638.
25. Segal B, Castro CB. Coral community structure and sedimentation at different distances from the coast of the Abrolhos bank, Brazil. Braz J Oceanogr. 2011; 59(2): 119-129.
26. Silva AS, Leão ZMAN, Kikuchi RKP, Costa AB, Souza JRB. Sedimentation in the coastal reefs of Abrolhos over the last decades. Cont Shelf Res. 2013; 70: 159-167. doi: 10.1016/j.csr.2013.06.002.
27. Marta-Almeida M, Mendes R, Amorim FN, Cirano M, Dias JM. Fundão dam colapse: oceanic dispersion of river Doce after the greatest Brazilian environmental accident. Mar Pollut Bull. 2016; 112: 359-364. doi: 10.1016/j.marpolbul.2016.07.039.
28. Miranda LS, Marques AC. Hidden impacts of the Samarco mining waste dam collapse to Brazilian marine fauna – an example from the staurozoans (Cnidaria). Biota Neotrop. 2016; 16(2): e20160169. doi: 10.1590/1676-0611-BN-2016-0169.
29. Rudorff N, Rudorff CM, Kampel M, Ortiz G. Remote sensing monitoring of the impact of a major mining wastewater disaster on the turbidity of the Doce River plume off the eastern Brazilian coast. ISPRS J Photogramm Remote Sens. 2018; 145: 349-361. doi: 10.1016/j.isprsjprs.2018.02.013.
30. Mazzei EF, Bertoncini AA, Pinheiro HT, Machado LF, Vilar CC, Guabiroba HC, et al. Newly discovered reefs in the southern Abrolhos Bank, Brazil: anthropogenic impacts and urgent conservation needs. Mar Pollut Bull. 2017; 114: 123-133. doi: 10.1016/j.marpolbul.2016.08.059.
31. Magris RA, Marta-Almeida M, Monteiro JA, Ban NC. A modelling approach to assess the impact of land mining on marine biodiversity: Assessment in coastal catchments experiencing catastrophic events (SW Brazil). Sci Total Environ. 2019; 659: 828-840. doi: 10.1016/j.scitotenv.2018.12.238.
32. Grilo CF, Quaresma VDS, Amorim GFL, Bastos AC. Changes in flocculation patterns of cohesive sediments after an iron ore mining dam failure. Mar Geol. 2018; 400: 1-11. doi: 10.1016/j.margeo.2018.03.004.
33. Queiroz HM, Nóbrega GN, Ferreira TO, Almeida LS, Romero TB, Santaella ST, et al. The Samarco mine tailing disaster: a possible time-bomb for heavy metals contamination? Sci Total Environ. 2018; 637: 498-506. doi: 10.1016/j.scitotenv.2018.04.370.
34. Hudspith M, Reichelt-Brushett A, Harrison PL. Factors affecting the toxicity of trace metals to fertilization success in broadcast spawning marine invertebrates: A review. Aquatic Toxicol. 2017; 184: 1-13. doi: 10.1016/j.aquatox.2016.12.019.
35. Freitas MO, Previero M, Leite JR, Francini-Filho RB, Minte-Vera CV, Moura RL. Age, growth, reproduction and management of Southwestern Atlantic’s largest and endangered herbivorous reef fish, *Scarus trispinosus* Valenciennes, 1840. PeerJ. 2019; 7: e7459. doi: 10.7717/peerj.7459.
36. Bueno, E. Capitães do Brasil: a saga dos primeiros colonizadores. Coleção Terra Brasilis; vol. III, Editora Objetiva Ltda., Rio de Janeiro, Brasil; 1999.
37. Morais IOB, Danilewicz D, Zerbini AN, Edmundson W, Hart IB, Bortolotto GA. From the southern right whale hunting decline to the humpback whaling expansion: a review of whale catch records in the tropical western South Atlantic Ocean. Mamm Rev. 2016. doi: 10.1111/mam.12073.
38. Freitas MO, Moura RL, Francini-Filho RB, Minte-Vera CV. Spawning patterns of comercially important reef fish (Lutjanidae and Serranidae) in the tropical western South Atlantic. Sci Mar. 2011; 75(1): 135-146. doi: 10.3989/scimar.2011.75n1135.
39. Aschenbrenner A, Freitas MO, Rocha GRA, Moura RL, Francini-Filho RB, Minte-Vera C, et al. Age, growth parameters, and fisheries índices for the lane snapper in the Abrolhos Bank, SW Atlantic. Fish Res. 2017; 194: 155-163. doi: 10.1016/j.fishres.2017.06.004.Francini-Filho RB, Moura RL. Dynamics of fish assemblages on coral reefs subjected to different management regimes in the Abrolhos Bank, eastern Brazil. Aquatic Conserv Mar Freshw Ecosyst. 2008; 18: 1166–1179. doi: 10.1002/aqc.966.Anderson MJ. A new method for non‐parametric multivariate analysis of variance. Austral Ecology 2001; 26: 32 - 46. doi: 10.1111/j.1442-9993.2001.01070.pp.x
40. Anderson MJ, Walsh DC. PERMANOVA, ANOSIM, and the Mantel test in the face of heterogeneous dispersions: what null hypothesis are you testing? Ecological Monographs 2013; 83: 557-574. doi: 10.1890/12-2010.1
