# Supplementary figures and images for: Decadal (2006-2018) dynamics of Southwestern Atlantic’s largest turbid zone reefs
Source: PLoS One. 2021 Feb 22;16(2):e0247111. doi: 10.1371/journal.pone.0247111 (PMC7899327; doi:10.1371/journal.pone.0247111)

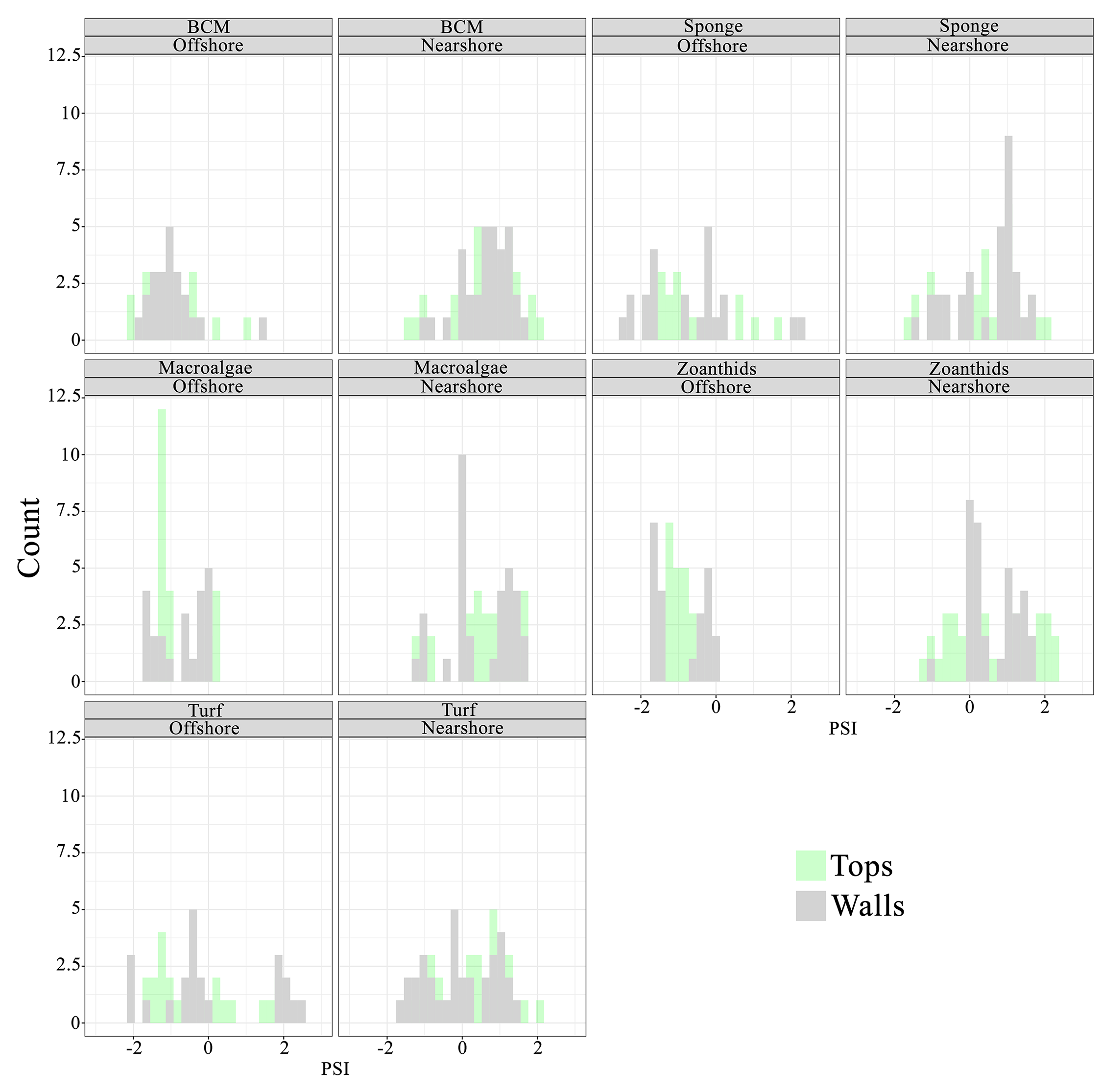

Supplement: S1 Fig — Frequency of Phase Shift Index (PSI) values for each main coral competitor at both arcs and habitats. BCM = benthic cyanobacteria mats. (TIF) [file pone.0247111.s003.tif]
