# Supplementary material for: Decadal (2006-2018) dynamics of Southwestern Atlantic’s largest turbid zone reefs
Source: PLoS One. 2021 Feb 22;16(2):e0247111. doi: 10.1371/journal.pone.0247111 (PMC7899327; doi:10.1371/journal.pone.0247111)
Supplement: S1 Table — Percent cover of the different groups for each year, site, and habitat sampled in the Abrolhos reefs between 2006 and 2018. (DOCX) [file pone.0247111.s004.docx]

**S1 Table. Summary of the dataset.**

| **Site** | **Habitat** | **Arc** | **Depth (m)** | **Year** | **Coral (%)** | **Hydrocoral (%)** | **CCA (%)** | **Sponge (%)** | **Zoanthids (%)** | **Macroalgae (%)** | **Turf (%)** | **BCM (%)** | **OO (%)** |
| --- | --- | --- | --- | --- | --- | --- | --- | --- | --- | --- | --- | --- | --- |
| PAB2 | W | O | 16.9 | 2006 | 7.5 | 0.0 | 0.1 | 0.8 | 0.0 | 0.0 | 74.5 | 16.8 | 0.3 |
|  |  |  |  | 2007 | 6.9 | 0.0 | 0.1 | 0.0 | 0.0 | 0.0 | 86.5 | 6.5 | 0.0 |
|  |  |  |  | 2008 | 7.8 | 0.1 | 10.0 | 1.4 | 0.0 | 0.5 | 52.0 | 24.9 | 3.3 |
|  |  |  |  | 2009 | 8.0 | 0.0 | 7.0 | 2.1 | 0.1 | 0.3 | 59.3 | 21.1 | 2.0 |
|  |  |  |  | 2012 | 8.0 | 0.0 | 11.6 | 1.4 | 0.1 | 0.7 | 60.2 | 14.6 | 3.3 |
|  |  |  |  | 2013 | 8.3 | 0.0 | 10.2 | 3.7 | 0.1 | 1.2 | 38.9 | 36.5 | 1.2 |
|  |  |  |  | 2014 | 7.6 | 0.0 | 12.6 | 2.0 | 0.0 | 0.8 | 50.1 | 24.8 | 2.1 |
|  |  |  |  | 2015 | 9.3 | 0.0 | 9.0 | 1.0 | 0.0 | 1.5 | 39.4 | 36.9 | 2.9 |
|  |  |  |  | 2016 | 7.5 | 0.0 | 11.4 | 1.7 | 0.0 | 1.9 | 41.9 | 32.7 | 2.8 |
|  |  |  |  | 2017 | 9.6 | 0.0 | 13.7 | 2.4 | 0.1 | 0.1 | 44.7 | 26.0 | 3.5 |
|  |  |  |  | 2018 | 9.1 | 0.0 | 15.2 | 2.9 | 0.0 | 0.5 | 39.0 | 31.5 | 1.7 |
| PAB3 | W | O | 17 | 2006 | 1.4 | 0.0 | 1.7 | 1.4 | 0.0 | 0.0 | 82.7 | 11.6 | 1.2 |
|  |  |  |  | 2007 | 0.4 | 0.0 | 1.6 | 0.0 | 0.0 | 0.0 | 93.4 | 4.6 | 0.1 |
|  |  |  |  | 2008 | 1.1 | 0.0 | 18.3 | 3.1 | 0.0 | 0.3 | 52.2 | 17.4 | 7.7 |
|  |  |  |  | 2009 | 0.8 | 0.0 | 8.2 | 6.4 | 0.0 | 0.0 | 75.1 | 7.5 | 1.9 |
|  |  |  |  | 2012 | 2.7 | 0.0 | 12.5 | 4.9 | 0.0 | 0.1 | 64.3 | 8.9 | 6.6 |
|  |  |  |  | 2013 | 3.0 | 0.0 | 4.8 | 2.7 | 0.0 | 0.9 | 64.9 | 19.7 | 4.0 |
|  |  |  |  | 2014 | 4.0 | 0.0 | 15.3 | 3.2 | 0.0 | 0.2 | 62.2 | 11.9 | 3.1 |
|  |  |  |  | 2015 | 2.3 | 0.0 | 12.2 | 4.6 | 0.0 | 1.0 | 64.0 | 12.9 | 3.0 |
|  |  |  |  | 2016 | 3.2 | 0.0 | 12.4 | 2.4 | 0.0 | 0.2 | 50.6 | 26.1 | 5.1 |
|  |  |  |  | 2017 | 1.6 | 0.0 | 12.1 | 2.7 | 0.0 | 0.3 | 60.4 | 18.1 | 4.9 |
|  |  |  |  | 2018 | 1.4 | 0.0 | 13.7 | 2.7 | 0.0 | 0.3 | 50.3 | 25.8 | 5.8 |
| PLES | W | N | 6.1 | 2006 | 36.1 | 0.0 | 13.3 | 0.2 | 0.1 | 0.3 | 44.3 | 5.8 | 0.0 |
| **Site** | **Habitat** | **Arc** | **Depth (m)** | **Year** | **Coral (%)** | **Hydrocoral (%)** | **CCA (%)** | **Sponge (%)** | **Zoanthids (%)** | **Macroalgae (%)** | **Turf (%)** | **BCM (%)** | **OO (%)** |
| PLES | W | N | 6.1 | 2007 | 32.4 | 0.0 | 12.6 | 0.8 | 0.0 | 0.0 | 51.4 | 2.5 | 0.3 |
|  |  |  |  | 2008 | 38.8 | 0.1 | 4.8 | 0.0 | 0.1 | 0.4 | 54.4 | 1.3 | 0.0 |
|  |  |  |  | 2009 | 32.7 | 0.0 | 10.0 | 0.5 | 0.2 | 0.0 | 53.5 | 3.0 | 0.1 |
|  |  |  |  | 2012 | 32.5 | 0.0 | 13.4 | 1.5 | 0.0 | 0.0 | 38.4 | 14.2 | 0.1 |
|  |  |  |  | 2013 | 30.4 | 0.0 | 6.4 | 0.9 | 0.1 | 0.0 | 34.2 | 27.7 | 0.2 |
|  |  |  |  | 2014 | 30.9 | 0.0 | 6.5 | 0.8 | 0.0 | 0.1 | 59.3 | 2.3 | 0.1 |
|  |  |  |  | 2015 | 31.1 | 0.0 | 11.0 | 1.1 | 0.1 | 0.1 | 51.5 | 5.1 | 0.0 |
|  |  |  |  | 2016 | 30.4 | 0.0 | 10.0 | 0.9 | 0.0 | 0.0 | 42.5 | 16.1 | 0.1 |
|  |  |  |  | 2017 | 30.3 | 0.0 | 9.1 | 0.7 | 0.1 | 0.1 | 50.9 | 8.7 | 0.1 |
|  |  |  |  | 2018 | 30.9 | 0.0 | 12.7 | 1.1 | 0.0 | 0.0 | 42.4 | 12.9 | 0.0 |
| SGOM | W | N | 8.2 | 2006 | 49.7 | 0.0 | 7.1 | 0.1 | 0.3 | 0.0 | 42.8 | 0.0 | 0.0 |
|  |  |  |  | 2007 | 43.1 | 0.0 | 2.6 | 0.0 | 0.1 | 0.0 | 53.8 | 0.4 | 0.0 |
|  |  |  |  | 2008 | 45.9 | 0.0 | 7.0 | 0.1 | 0.3 | 0.2 | 45.9 | 0.6 | 0.0 |
|  |  |  |  | 2009 | 43.1 | 0.0 | 0.2 | 0.0 | 0.4 | 0.0 | 55.5 | 0.8 | 0.0 |
|  |  |  |  | 2010 | 47.7 | 0.0 | 1.4 | 0.1 | 0.5 | 0.0 | 38.4 | 11.8 | 0.1 |
|  |  |  |  | 2012 | 54.3 | 0.0 | 5.0 | 0.0 | 1.1 | 0.0 | 38.8 | 0.7 | 0.1 |
|  |  |  |  | 2013 | 50.7 | 0.0 | 0.4 | 0.0 | 0.7 | 0.0 | 32.8 | 15.4 | 0.0 |
|  |  |  |  | 2014 | 56.6 | 0.0 | 3.7 | 0.2 | 0.8 | 0.0 | 35.7 | 2.9 | 0.1 |
|  |  |  |  | 2016 | 54.7 | 0.0 | 3.7 | 0.1 | 0.2 | 0.0 | 39.4 | 1.9 | 0.0 |
|  |  |  |  | 2017 | 56.5 | 0.0 | 1.1 | 0.1 | 0.4 | 0.0 | 38.0 | 3.9 | 0.0 |
|  |  |  |  | 2018 | 57.5 | 0.0 | 3.8 | 0.1 | 0.2 | 0.1 | 34.2 | 4.0 | 0.0 |
| TIMB | W | N | 10.5 | 2006 | 13.4 | 0.2 | 18.9 | 0.1 | 2.3 | 4.6 | 48.2 | 12.1 | 0.2 |
|  |  |  |  | 2007 | 12.2 | 0.3 | 15.0 | 1.1 | 2.4 | 4.3 | 57.2 | 7.3 | 0.3 |
|  |  |  |  | 2008 | 14.5 | 0.3 | 31.1 | 1.7 | 2.4 | 5.2 | 40.8 | 4.0 | 0.0 |
|  |  |  |  | 2009 | 12.8 | 0.2 | 13.5 | 1.5 | 1.8 | 2.1 | 61.6 | 5.7 | 0.8 |
| **Site** | **Habitat** | **Arc** | **Depth (m)** | **Year** | **Coral (%)** | **Hydrocoral (%)** | **CCA (%)** | **Sponge (%)** | **Zoanthids (%)** | **Macroalgae (%)** | **Turf (%)** | **BCM (%)** | **OO (%)** |
| TIMB | W | N | 10.5 | 2010 | 13.7 | 0.3 | 17.5 | 3.0 | 1.8 | 2.8 | 52.8 | 8.0 | 0.1 |
|  |  |  |  | 2012 | 8.5 | 0.2 | 24.7 | 2.3 | 1.6 | 2.7 | 50.0 | 9.8 | 0.2 |
|  |  |  |  | 2013 | 7.9 | 0.1 | 17.1 | 1.8 | 1.8 | 2.0 | 48.4 | 20.9 | 0.1 |
|  |  |  |  | 2014 | 7.9 | 0.0 | 29.7 | 2.6 | 1.8 | 2.3 | 47.7 | 7.9 | 0.2 |
|  |  |  |  | 2015 | 9.5 | 0.1 | 28.6 | 2.5 | 1.3 | 2.1 | 50.3 | 5.5 | 0.1 |
|  |  |  |  | 2016 | 8.0 | 0.2 | 23.6 | 2.8 | 1.5 | 3.9 | 52.5 | 7.3 | 0.1 |
|  |  |  |  | 2017 | 8.0 | 0.2 | 20.8 | 3.6 | 1.7 | 2.4 | 53.8 | 9.4 | 0.1 |
| PAB2 | T | O | 7.3 | 2006 | 16.0 | 0.6 | 2.8 | 0.0 | 2.9 | 0.0 | 71.3 | 6.5 | 0.0 |
|  |  |  |  | 2007 | 11.8 | 0.7 | 0.6 | 0.0 | 1.7 | 0.0 | 84.2 | 1.1 | 0.0 |
|  |  |  |  | 2008 | 16.4 | 0.6 | 5.7 | 0.2 | 1.4 | 0.0 | 58.4 | 15.6 | 1.6 |
|  |  |  |  | 2009 | 15.7 | 0.7 | 1.2 | 0.2 | 2.0 | 0.1 | 68.2 | 11.8 | 0.1 |
|  |  |  |  | 2012 | 16.1 | 0.4 | 1.4 | 0.0 | 0.7 | 0.1 | 61.9 | 19.2 | 0.1 |
|  |  |  |  | 2013 | 14.7 | 0.3 | 5.3 | 0.0 | 1.6 | 0.0 | 53.4 | 24.6 | 0.0 |
|  |  |  |  | 2014 | 17.3 | 0.7 | 2.6 | 0.2 | 0.8 | 0.1 | 65.0 | 13.2 | 0.1 |
|  |  |  |  | 2015 | 15.9 | 0.6 | 2.8 | 0.2 | 0.9 | 0.0 | 59.2 | 20.2 | 0.2 |
|  |  |  |  | 2016 | 17.2 | 0.7 | 1.8 | 0.3 | 1.7 | 0.0 | 72.1 | 5.8 | 0.4 |
|  |  |  |  | 2017 | 18.7 | 0.2 | 4.0 | 0.3 | 1.0 | 0.1 | 67.2 | 7.9 | 0.8 |
|  |  |  |  | 2018 | 20.6 | 0.3 | 5.7 | 0.4 | 1.8 | 0.0 | 56.1 | 14.8 | 0.3 |
| PAB3 | T | O | 6.9 | 2006 | 13.1 | 0.5 | 10.6 | 0.1 | 10.1 | 0.0 | 63.4 | 2.2 | 0.0 |
|  |  |  |  | 2007 | 11.9 | 0.3 | 5.9 | 0.0 | 10.7 | 0.0 | 65.5 | 5.7 | 0.0 |
|  |  |  |  | 2008 | 14.9 | 0.6 | 15.1 | 0.1 | 9.6 | 0.0 | 55.3 | 4.3 | 0.1 |
|  |  |  |  | 2009 | 14.2 | 0.4 | 4.4 | 0.0 | 9.2 | 0.0 | 60.1 | 11.6 | 0.2 |
|  |  |  |  | 2012 | 13.2 | 0.7 | 5.0 | 0.1 | 0.9 | 0.0 | 75.3 | 4.5 | 0.2 |
|  |  |  |  | 2013 | 14.2 | 1.1 | 3.3 | 0.1 | 3.1 | 0.1 | 62.8 | 15.3 | 0.1 |
|  |  |  |  | 2014 | 13.6 | 0.9 | 8.1 | 0.1 | 4.0 | 0.0 | 57.4 | 15.5 | 0.4 |
| **Site** | **Habitat** | **Arc** | **Depth (m)** | **Year** | **Coral (%)** | **Hydrocoral (%)** | **CCA (%)** | **Sponge (%)** | **Zoanthids (%)** | **Macroalgae (%)** | **Turf (%)** | **BCM (%)** | **OO (%)** |
| PAB3 | T | O | 6.9 | 2015 | 15.3 | 1.0 | 9.4 | 0.0 | 5.1 | 0.8 | 65.4 | 2.7 | 0.2 |
|  |  |  |  | 2016 | 16.6 | 1.2 | 6.5 | 0.3 | 4.0 | 0.1 | 67.6 | 3.4 | 0.3 |
|  |  |  |  | 2017 | 17.3 | 0.9 | 4.4 | 0.2 | 4.4 | 0.0 | 66.8 | 5.8 | 0.2 |
|  |  |  |  | 2018 | 16.4 | 0.9 | 6.5 | 0.3 | 4.5 | 0.0 | 62.8 | 8.5 | 0.3 |
| PLES | T | N | 2.3 | 2006 | 11.9 | 1.1 | 5.6 | 0.0 | 0.1 | 44.1 | 36.7 | 0.4 | 0.2 |
|  |  |  |  | 2007 | 6.9 | 1.5 | 1.9 | 0.0 | 0.1 | 39.7 | 41.4 | 8.4 | 0.2 |
|  |  |  |  | 2008 | 3.7 | 1.6 | 0.8 | 0.0 | 0.0 | 75.4 | 18.2 | 0.0 | 0.3 |
|  |  |  |  | 2009 | 9.3 | 1.2 | 0.6 | 0.0 | 0.2 | 34.3 | 50.8 | 3.2 | 0.3 |
|  |  |  |  | 2012 | 6.6 | 1.4 | 2.0 | 0.0 | 0.0 | 33.5 | 53.4 | 2.6 | 0.4 |
|  |  |  |  | 2013 | 4.4 | 1.3 | 1.5 | 0.0 | 0.0 | 28.8 | 52.4 | 11.4 | 0.2 |
|  |  |  |  | 2014 | 8.9 | 2.1 | 1.5 | 0.1 | 0.0 | 25.4 | 60.6 | 1.3 | 0.1 |
|  |  |  |  | 2015 | 10.1 | 2.0 | 1.2 | 0.0 | 0.0 | 25.5 | 59.9 | 1.0 | 0.2 |
|  |  |  |  | 2016 | 10.0 | 2.2 | 1.2 | 0.1 | 0.0 | 14.2 | 59.2 | 12.9 | 0.1 |
|  |  |  |  | 2017 | 11.3 | 0.6 | 0.6 | 0.0 | 0.1 | 24.5 | 60.2 | 2.4 | 0.3 |
|  |  |  |  | 2018 | 12.6 | 0.5 | 1.3 | 0.0 | 0.0 | 11.4 | 73.2 | 0.9 | 0.1 |
| SGOM | T | N | 3.2 | 2006 | 3.4 | 0.7 | 2.0 | 0.1 | 31.1 | 4.4 | 57.3 | 0.6 | 0.5 |
|  |  |  |  | 2007 | 3.5 | 0.3 | 0.6 | 0.0 | 18.9 | 31.5 | 44.2 | 0.0 | 0.8 |
|  |  |  |  | 2008 | 3.3 | 0.4 | 1.7 | 0.0 | 21.1 | 14.8 | 57.8 | 0.0 | 1.0 |
|  |  |  |  | 2009 | 3.4 | 0.3 | 0.5 | 0.0 | 21.3 | 9.3 | 62.5 | 1.8 | 0.8 |
|  |  |  |  | 2010 | 4.6 | 0.2 | 1.1 | 0.1 | 25.6 | 0.3 | 64.9 | 2.0 | 1.2 |
|  |  |  |  | 2012 | 4.4 | 0.0 | 1.7 | 0.0 | 25.2 | 4.3 | 63.1 | 0.0 | 1.3 |
|  |  |  |  | 2013 | 3.4 | 0.0 | 0.2 | 0.0 | 22.3 | 11.0 | 57.5 | 3.3 | 2.2 |
|  |  |  |  | 2014 | 4.6 | 0.1 | 2.4 | 0.2 | 25.8 | 1.9 | 62.4 | 0.1 | 2.6 |
|  |  |  |  | 2016 | 7.2 | 0.4 | 2.0 | 0.0 | 30.1 | 0.2 | 55.4 | 3.2 | 1.6 |
|  |  |  |  | 2017 | 5.5 | 0.4 | 0.6 | 0.0 | 33.6 | 11.5 | 46.0 | 0.3 | 2.0 |
| **Site** | **Habitat** | **Arc** | **Depth (m)** | **Year** | **Coral (%)** | **Hydrocoral (%)** | **CCA (%)** | **Sponge (%)** | **Zoanthids (%)** | **Macroalgae (%)** | **Turf (%)** | **BCM (%)** | **OO (%)** |
| SGOM | T | N | 3.2 | 2018 | 5.3 | 0.5 | 0.8 | 0.0 | 32.0 | 0.7 | 55.8 | 1.3 | 3.7 |
| TIMB | T | N | 5.6 | 2006 | 4.2 | 1.0 | 14.5 | 0.0 | 26.5 | 5.6 | 45.8 | 2.4 | 0.0 |
|  |  |  |  | 2007 | 3.7 | 0.4 | 4.6 | 0.0 | 27.7 | 6.9 | 55.1 | 1.6 | 0.0 |
|  |  |  |  | 2008 | 3.5 | 1.2 | 6.5 | 0.0 | 28.2 | 7.6 | 48.4 | 4.6 | 0.0 |
|  |  |  |  | 2009 | 3.8 | 0.3 | 2.3 | 0.0 | 28.6 | 3.8 | 59.7 | 1.5 | 0.0 |
|  |  |  |  | 2010 | 4.8 | 0.2 | 11.5 | 0.0 | 22.6 | 6.3 | 51.9 | 2.6 | 0.2 |
|  |  |  |  | 2012 | 5.0 | 0.1 | 6.9 | 0.0 | 18.0 | 4.0 | 59.9 | 6.0 | 0.2 |
|  |  |  |  | 2013 | 5.7 | 0.2 | 3.2 | 0.1 | 12.1 | 7.6 | 54.5 | 16.5 | 0.0 |
|  |  |  |  | 2014 | 6.8 | 0.3 | 11.7 | 0.0 | 10.5 | 6.2 | 59.4 | 5.1 | 0.0 |
|  |  |  |  | 2015 | 8.0 | 0.4 | 13.1 | 0.1 | 8.8 | 8.1 | 59.5 | 2.1 | 0.1 |
|  |  |  |  | 2016 | 7.7 | 0.8 | 7.4 | 0.0 | 9.8 | 18.7 | 53.2 | 2.2 | 0.0 |
|  |  |  |  | 2017 | 7.5 | 0.5 | 11.0 | 0.0 | 9.7 | 10.5 | 53.2 | 7.5 | 0.0 |

CCA = crustose calcareous algae, BCM = benthic cyanobacteria mats, OO = other organisms, W = Wall, T = Top, O = Offshore, N = Nearshore, PAB2 and PAB3 = Parcel dos Abrolhos 2 and 3, PLES = Pedra de Leste reef, SGOM = Sebastião Gomes reef, TIMB = Timbebas reef.
